# Regulation of zebrafish dorsoventral patterning by phase separation of RNA-binding protein Rbm14

**DOI:** 10.1038/s41421-019-0106-x

**Published:** 2019-07-23

**Authors:** Yue Xiao, Jiehui Chen, Yihan Wan, Qi Gao, Naihe Jing, Yixian Zheng, Xueliang Zhu

**Affiliations:** 1 State Key Laboratory of Cell Biology, CAS Center for Excellence in Molecular Cell Science, Shanghai Institute of Biochemistry and Cell Biology, Chinese Academy of Sciences, University of Chinese Academy of Sciences, 320 Yueyang Road, 200031 Shanghai, China; 2 Department of Embryology, Carnegie Institution for Science, 3520 San Martin Dr., Baltimore, MD 21218 USA

**Keywords:** Cell signalling, Developmental biology

## Abstract

RNA-binding proteins with intrinsically disordered regions (IDRs) such as Rbm14 can phase separate in vitro. To what extent the phase separation contributes to their physiological functions is however unclear. Here we show that zebrafish Rbm14 regulates embryonic dorsoventral patterning through phase separation. Zebrafish *rbm14* morphants displayed dorsalized phenotypes associated with attenuated BMP signaling. Consistently, depletion of mammalian Rbm14 downregulated BMP regulators and effectors Nanog, Smad4/5, and Id1/2, whereas overexpression of the BMP-related proteins in the morphants significantly restored the developmental defects. Importantly, the IDR of zebrafish Rbm14 demixed into liquid droplets in vitro despite poor sequence conservation with its mammalian counterpart. While its phase separation mutants or IDR failed to rescue the morphants, its chimeric proteins containing an IDR from divergent phase separation proteins were effective. Rbm14 complexed with proteins involved in RNA metabolism and phase separated into cellular ribonucleoprotein compartments. Consistently, RNA deep sequencing analysis on the morphant embryos revealed increased alternative splicing events as well as large-scale transcriptomic downregulations. Our results suggest that Rbm14 functions in ribonucleoprotein compartments through phase separation to modulate multiple aspects of RNA metabolism. Furthermore, IDRs conserve in phase separation ability but not primary sequence and can be functionally interchangeable.

## Introduction

Proteins containing intrinsically disordered regions (IDRs) including the prion-like domains (PLDs)^1^ and the more general low complexity regions have been shown to demix or phase separate (or coacervate) from their aqueous solutions into supramolecular condensates such as liquid droplets and hydrogels in vitro mainly in an aromatic amino acids (especially Y residues)-dependent manner^2–7^. IDR-containing proteins display divergent phase separation properties, demixing at dramatically different critical concentrations and forming liquid droplets of varying dynamics and rigidity^7–9^. Multiple proteins can also co-phase separate, often in synergy^10,11^.

Although IDRs are poorly conserved in sequence even among orthologues, their amino acid compositions have been shown to affect their phase properties in vitro. For instance, the heterogeneous nuclear ribonucleoprotein (hnRNP) family members FUS, EWSR1, TAF15, and hnRNPA1, which contain a PLD and 1–2 RNA-recognition motifs (RRMs), phase separate at dramatically different critical concentrations^10,12^. Systematic studies mainly on FUS further suggest that the intermolecular interactions between aromatic and basic amino acid residues (especially between Y and R) largely decide the critical concentration, whereas G, Q, and S residues contribute to physical properties^10^. These in vitro studies imply that coacervates formed by different IDRs exhibit different physical properties that could be important for biological functions. On the other hand, as RNA granules often contain multiple players capable of the synergetic co-phase separation^2,10,13–15^, IDRs in different proteins might be at least partially interchangeable in vivo.

Accumulating evidence supports the role of protein phase separation in a variety of cellular activities^3,7,8,11,15–17^. For instance, phase separation properties of hnRNP proteins are essential to the formation of RNA granules including stress granules (SGs) and paraspeckles^6,15,18–20^. Mutations affecting their phase properties are shown to be associated with neurodegenerative diseases^21–26^. Nevertheless, as mutants containing numerous changes (usually Y-to-S mutations) are commonly used to address the importance of phase separation in vivo, it is almost impossible to ensure that the mutations do not affect other functions of the proteins irrelevant to phase separation.

Human Rbm14 (also called CoAA for Co-Activator Activator) contains two RRMs followed by a C-terminal PLD that can undergo phase separation to form hydrogels^6,27^. It is implicated in cell differentiation as a long non-coding RNA (lncRNA)-binding protein and regulator of gene transcription and pre-mRNA splicing^27–31^. It is also an essential component of paraspeckles, punctate nuclear compartments capable of retaining certain mRNAs and consequently regulating their transcription and stability, formed by the paraspeckle-specific lncRNA *Neat1*-induced protein phase separation^6,20,32–36^. Despite of these studies, it remains unclear how Rbm14 would function in embryonic development and whether its functions rely on its phase separation property.

In this study, we report an important role of Rbm14 in the embryonic dorsoventral patterning by using zebrafish as a model system. We show that Rbm14 functions in RNP compartments through phase separation to regulate RNA metabolism. Furthermore, our results indicate that the IDR of zebrafish Rbm14 (zRbm14) can be functionally replaced in vivo by IDRs from other proteins known to undergo phase separation.

## Results

### Zebrafish *rbm14* is highly expressed in early embryos

**Fig. 1:**
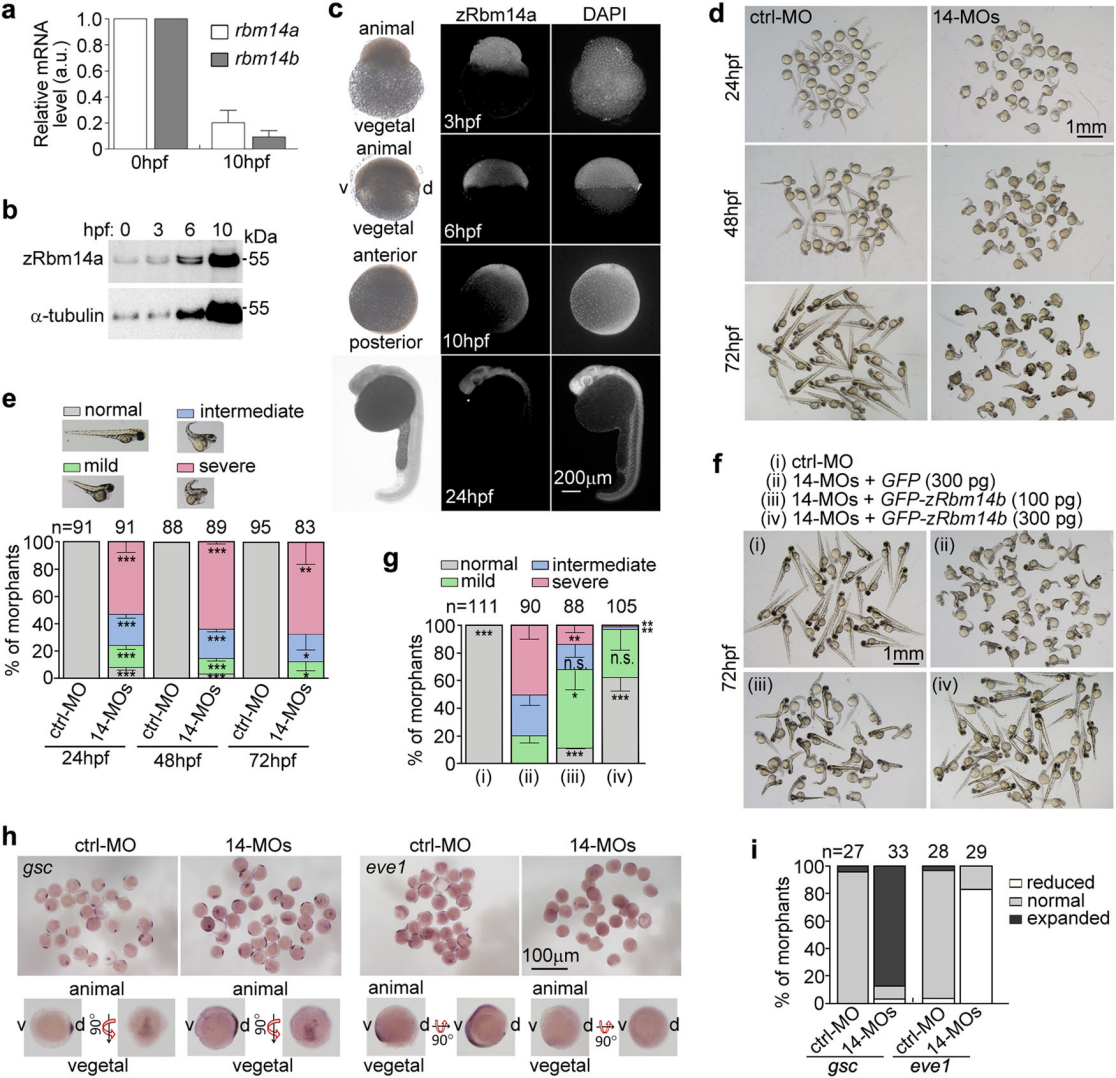
Zebrafish *rbm14* morphants display dorsalized phenotypes. **a** *rbm14* was downregulated during embryogenesis. Total mRNAs were extracted from 50 zebrafish embryos at 0 or 10 hpf. The expression levels of *rbm14a* and *rbm14b* were analyzed by qPCR. β-Actin was used as internal control. qPCR results from two independent experiments are presented as mean ± SD. **b** Total protein levels of zRbm14a increased during early embryonic development. Zebrafish embryos at the indicated stages were removed of the yolk and subjected to immunoblotting. Proteins from 8 embryos were loaded in each lane. **c** Immunofluorescent staining of zRbm14 in zebrafish embryos. Nuclear DNA was stained by DAPI. The animal and vegetal poles and ventral (v) and dorsal (d) sides are marked in the bright-field images. **d** Embryo morphologies at the indicated developmental stages. Zebrafish embryos at the one-cell stage were injected with a control morpholino oligonucleotide (ctrl-MO; 8 ng per embryo) or two MOs specific to *rbm14a* and *rbm14b*, respectively (14-MOs; 4 ng each per embryo) (see Supplementary Fig. 2a–e). **e** Quantification results for the experiments in **d**, based on the criteria and examples shown. **f**, **g** Exogenous zRbm14b rescued the dorsalized phenotypes. Zebrafish embryos at the one-cell stage were co-injected with the indicated MOs (total 8 ng per embryo) and in vitro-transcribed mRNA (also see Supplementary Fig. 1f). The morphants were examined at 72 hpf. Those injected with ctrl-MO served as negative control. Data in **e** and **g**, presented as mean ± SD, were from three independent experiments. Student’s *t*-test against the ctrl-MO-injected populations: n.s., no significance (*P* > 0.05); *P < 0.05; **P < 0.01; ***P < 0.001. Total numbers of embryos analyzed are listed over histograms. **h**, **i** The *rbm14* morphants show defective dorsoventral patterning. Zebrafish embryos microinjected as in **d** were subjected to mRNA in situ hybridization at the 75–90% epiboly stages (**h**) and quantified (**i**). Total numbers of embryos analyzed are listed over each histogram

There are two *Rbm14*-homologous genes in zebrafish, *zgc110682* (termed herein as *rbm14a*) and *zgc85696* (*rbm14b*). Their encoded proteins, zRbm14a and zRbm14b, share an identity of 49% with each other and of 47% and 34%, respectively, with mouse Rbm14 (mRbm14). Quantitative real-time PCR (qPCR) analyses showed that their mRNA levels were high in fertilized eggs but became concomitantly downregulated by >5-fold at the bud stage at 10 h post fertilization (hpf) (Fig. 1a)^37^. The total protein levels of zRbm14a per embryo markedly increased from 0 to 10 hpf (Fig. 1b). Cellular zRbm14a was highly concentrated at the animal pole at 3 hpf and relatively abundant in the ventral-animal and dorsal-animal areas at 6 hpf (Fig. 1c). It was highly expressed in the anterior region at 10 and 24 hpf (Fig. 1c). Thus, *rbm14a* and *rbm14b* are maternally expressed genes and may have a role in early embryonic development.

### *rbm14a* and *rbm14b* are functionally redundant

To assess their functions, we designed two antisense morpholino oligonucleotides (14a-MO and 14b-MO) to respectively block the translation initiation site of the *rbm14a* mRNA and a splicing site of the *rbm14b* pre-mRNA that would cause a reading frame shift (Supplementary Fig. 1a). Immunoblotting or reverse transcriptase PCR (RT-PCR) verified that 14a-MO did not affect the mRNA levels of the two genes but markedly downregulated the protein levels of zRbm14a, whereas 14b-MO specifically blocked the correct splicing of *rbm14b* mRNA in zebrafish embryos (Supplementary Fig. 1b, c).

Similar to the control MO (ctrl-MO), microinjection of 14b-MO into one-cell-stage zebrafish embryos at 4 ng per embryo had no obvious effects on the embryonic development when examined at 72 hpf (Supplementary Fig. 1d). By contrast, 14a-MO at the same dosage resulted in mild abnormalities in trunk and tail development and yolk extension (Supplementary Fig. 1d). Microinjecting both MOs (14-MOs) at a total of 4 ng per embryo (i.e., 2 ng of each MO), however, resulted in more severe defects, generating larva with shorter distorted posterior trunk and tail and with reduced yolk extension (Supplementary Fig. 1d), suggesting a functional redundancy of the two paralogous genes. Furthermore, zebrafish embryos injected with a total of 8 ng per embryo of 14-MOs manifested even stronger abnormalities, indicating a dose-dependent effect (Supplementary Fig. 1e). As 14-MOs injected at a total of 16 ng per embryo resulted in severe death at 72 hpf, we used 8 ng per embryo as the optimal dosage for subsequent experiments and termed the embryos *rbm14* morphants.

### Zebrafish *rbm14* morphants display dorsalized phenotypes

We found that the developmental abnormalities in the *rbm14* morphants were readily observed from 24 to 72 hpf (Fig. 1d). Furthermore, these abnormalities are highly similar to the dorsalized phenotypes reported in previous studies^38–41^. When the embryos were divided into four groups according to the severity of the abnormality (normal, mild, intermediate, and severe), over 88% of the *rbm14* morphants at 72 hpf were obviously (severe+intermediate) dorsalized (Fig. 1e).

To rule out off-target effect we performed rescue experiments by co-injecting in vitro-transcribed *GFP* or *GFP-zRbm14b* mRNA with 14-MOs. The fluorescence of GFP was observed at 10 hpf, indicating expression of the proteins (Supplementary Fig. 1f). Although GFP-zRbm14b was weakly expressed at 10 hpf as compared to GFP alone (Supplementary Fig. 1f), it significantly rescued the dorsalized phenotypes when examined at 72 hpf (Fig. 1f, g). In the *rbm14* morphants injected with 300 pg *GFP* mRNA per embryo, 80% of the embryos at 72 hpf were still obviously (severe+intermediate) dorsalized. By sharp contrast, *GFP-zRbm14b* mRNA injected at 100 or 300 pg per embryo reduced the incidence to 32% and 3%, respectively (Fig. 1f, g). Thus the dorsalized phenotype is caused by the loss of zRbm14.

To clarify whether the dorsalized phenotypes attributed to impaired ventralization during early embryonic development, we examined expression patterns of typical dorsoventral markers at 75–90% epiboly (8–9 hpf) through in situ hybridization^37^. Compared to the control morphants, the *rbm14* morphants displayed an expanded expression of the dorsal organizer *goosecoid* (*gsc*)^42^ and a reduced expression of the ventral marker and BMP target *even-skipped 1* (*eve1*)^43^ (Fig. 1h, i). Thus, Rbm14 is an important ventralization factor.

### Rbm14 is important for BMP signaling by maintaining the levels of multiple BMP effectors

**Fig. 2:**
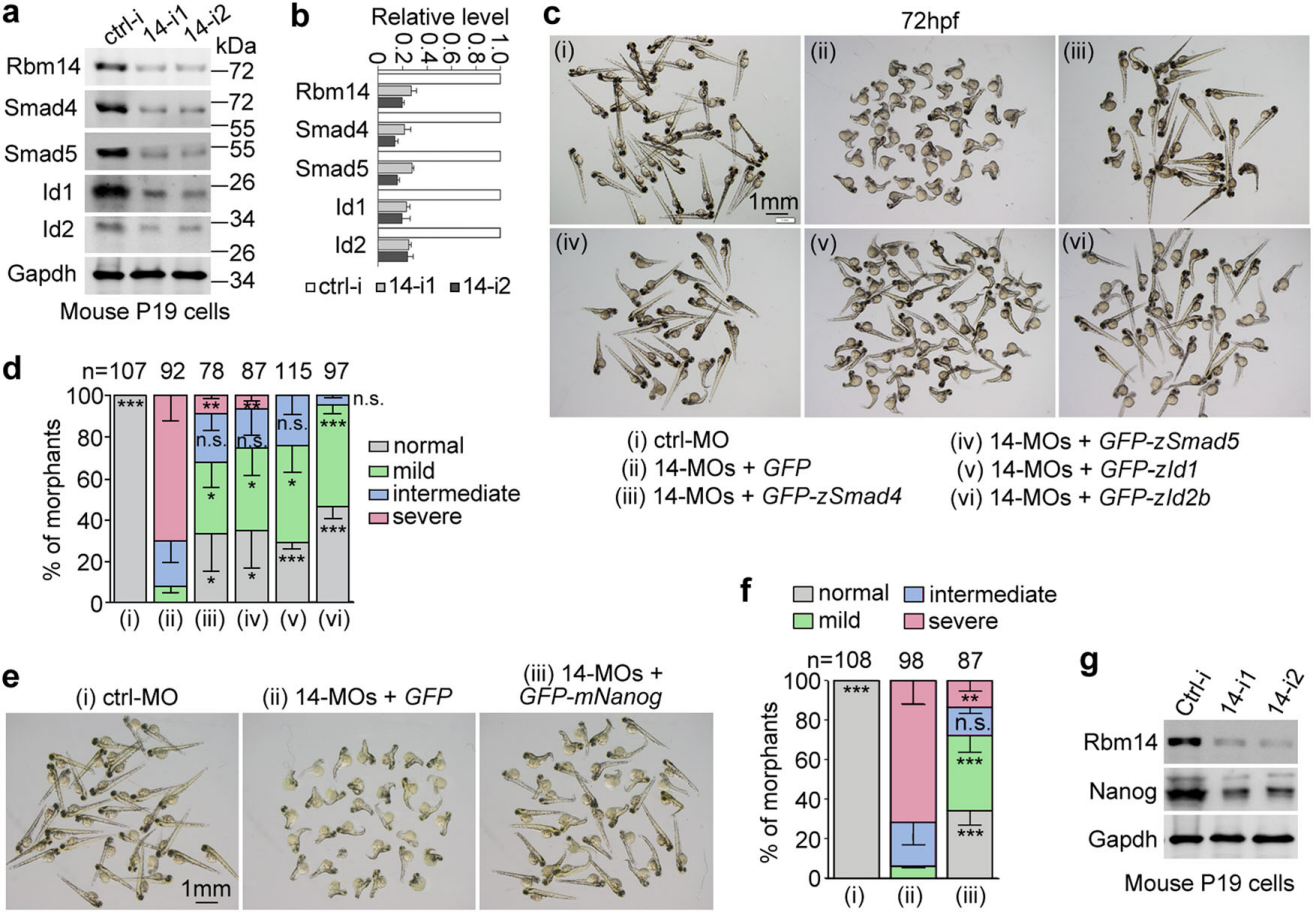
The ventralization defects of *rbm14* morphants is mainly attributed to insufficient BMP signaling. **a**, **b** Knockdown of mRbm14 in P19 cells concomitantly downregulated Smad4, Smad5, Id1, and Id2. P19 cells transfected with control siRNA (ctrl-i) or each of the two *mRbm14*-specific siRNAs (14-i1 and 14-i2) for 48 h were collected and subjected to immunoblotting. Gapdh served as a loading control. The quantification results (**b**), presented as mean ± SD, were based on band intensities from two independent experiments. **c**, **d** Overexpressing smad4, smad5, id1, or id2b attenuated the ventralization defects of *rbm14* morphants. Zebrafish embryos at the one-cell stage were co-injected with the indicated MOs (total 8 ng per embryo) and in vitro-transcribed mRNA (300 pg per embryo) coding for GFP or the GFP-tagged proteins (also see Supplementary Fig. 2). Those injected with ctrl-MO served as a negative control. **e**, **f** Overexpressing Nanog attenuates the ventralization defect of zebrafish *rbm14* morphants. Zebrafish embryos at the one-cell stage were co-injected with the indicated MOs (total 8 ng per embryo) and in vitro-transcribed mRNA coding for GFP or GFP-mNanog (300 pg per embryo) (also see Supplementary Fig. 2). Embryos injected with ctrl-MO served as a negative control. Quantification results (**d**, **f**), based on the criteria and examples in Fig. 1e and presented as mean ± SD, were from three independent experiments. Student’s *t*-test against the GFP mRNA-injected populations: n.s., no significance (*P* > 0.05); *P < 0.05; **P < 0.01; ***P < 0.001. Total number of embryos analyzed are listed over each histogram. **g** Depletion of Rbm14 in P19 cells downregulated Nanog. P19 cells transfected with the indicated siRNAs for 48 h were subjected to immunoblotting. Gapdh served as a loading control

BMP signaling plays a key role in vertebrate dorsal–ventral patterning during gastrulation^44–46^. Its inhibition usually results in dorsalized patterning of zebrafish body axis^38–41,47,48^. We thus investigated whether Rbm14 could affect the BMP pathway. Due to the limitation on available antibodies against zebrafish proteins and the lack of zebrafish cell lines for such analysis, we silenced *mRbm14* by RNAi in the mouse pluripotent P19 cells. Interestingly, we found that key components of the BMP pathway, Smad4 and Smad5, and the downstream effectors Id1 and Id2 were markedly downregulated (Fig. 2a, b)^49,50^.

Next we explored whether supplementing the zSmad or zId proteins could attenuate the severity of the dorsalized phenotypes. When 300 pg of in vitro-transcribed mRNA were co-injected with 14-MOs to express GFP or GFP-tagged zSmad4, zSmad5, zId1, or zId2b (Supplementary Fig. 2), we found that, compared to GFP alone, all the GFP-tagged proteins significantly decreased the abnormality of the *rbm14* morphants: the majority of the fish expressing the BMP pathway proteins displayed elongated body axis similar to the ctrl-MO-injected fish (Fig. 2c, d). Thus, Rbm14 maintains the levels of multiple BMP effectors to sustain the strength of BMP signaling in both mouse cells and zebrafish.

### Nanog functions downstream of Rbm14 in zebrafish embryos

The homeoprotein Nanog is critical for the ventralization of zebrafish embryos by activating the BMP signaling. Zebrafish *nanog* morphants thus displayed dorsalized phenotypes^51^. To understand the relationship between Rbm14 and Nanog, we microinjected 300 pg of in vitro-transcribed *GFP-mNanog* mRNA with 14-MOs and observed obvious rescue effects as compared to *GFP* mRNA alone (Fig. 2e, f; Supplementary Fig. 2). These results suggest Nanog as a downstream target of Rbm14.

We then examined Rbm14-depleted P19 cells and found that Nanog was downregulated as well (Fig. 2g). Therefore, Rbm14 is also important for the maintenance of Nanog levels in mammalian cells.

### Zebrafish Rbm14 displays punctate subcellular distributions

**Fig. 3:**
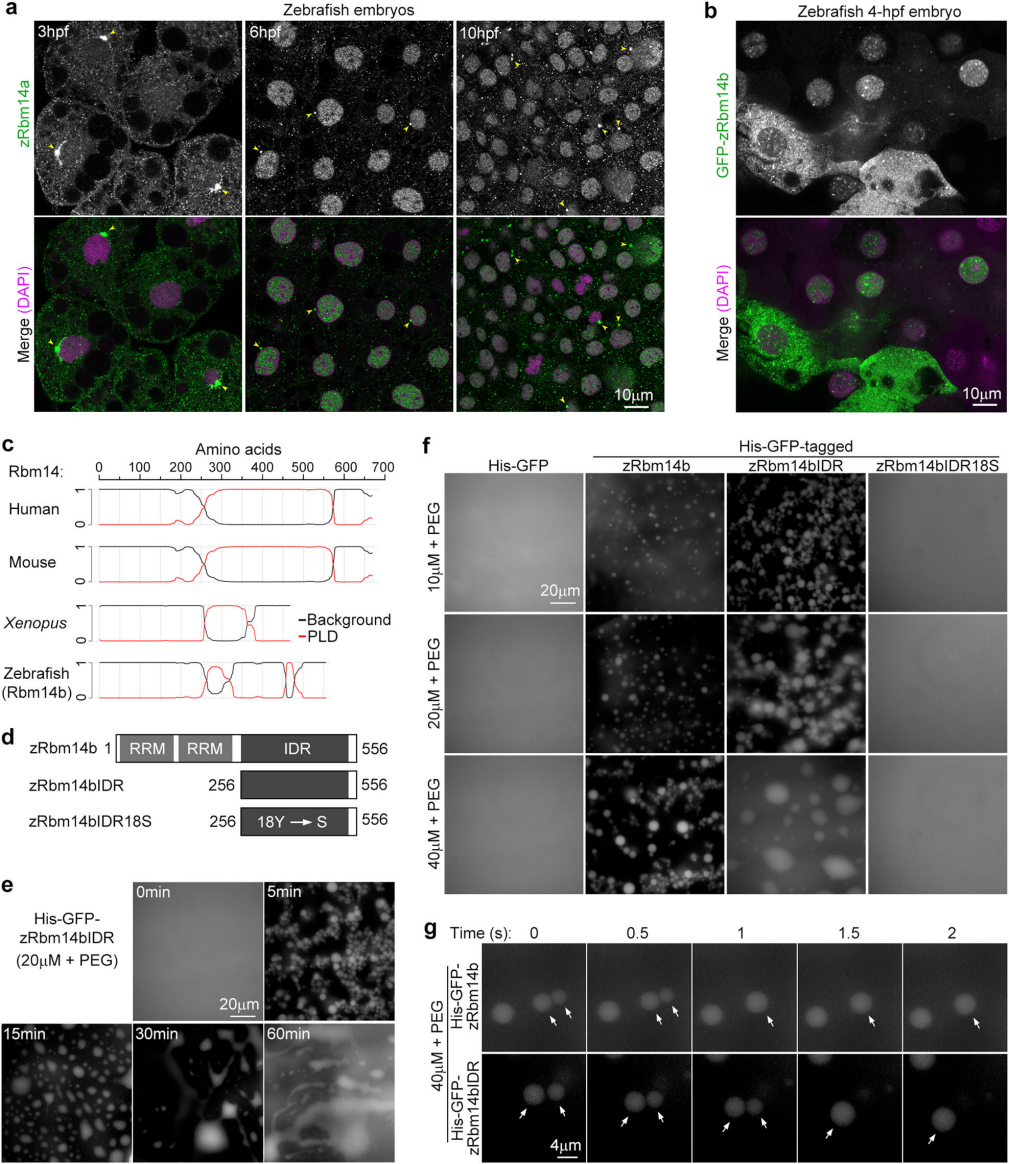
zRbm14 distributes in cells as puncta and forms liquid droplets in vitro through IDR. **a** zRbm14a displayed punctate distributions in the cytoplasm and nucleus of zebrafish embryonic cells. Zebrafish embryos immunostained as in Fig. 1c were imaged at high resolution. Arrowheads indicate bright perinuclear zRbm14a puncta. **b** GFP-zRbm14b displayed similar subcellular localizations as endogenous zRbm14a. Zebrafish embryos were microinjected at the one-cell stage with in vitro-transcribed mRNA (800 pg per embryo) to express GFP-zRbm14b, fixed at approximately 4 hpf, and imaged at high resolution. **c** PLD prediction for Rbm14 orthologues. The diagrams were generated by using the Prion-like Amino Acid Composition (PLAAC) program (http://plaac.wi.mit.edu)^1^. Sequences with the PLD probability >0.5 (*y-*axis) are considered as a PLD. **d** Diagrams of His-GFP-tagged zRbm14b and its mutants. Detailed mutation sites in zRbm14bIDR18S are indicated in Supplementary Fig. 3a. **e** Time-dependent droplet growth. Twenty micromolar of His-GFP-zRbm14bIDR containing 1% PEG8000 were incubated at 25 °C for the indicated time and imaged for GFP fluorescence. **f** Concentration-dependent droplet formation. Ten to 40 μM of purified His-GFP or its tagged proteins containing 1% PEG8000 were shifted from ice to 25 °C for 5 min and imaged. Also see Supplementary Fig. 3b, c. **g** Image sequences showing fusion processes of two droplets (arrows)

To gain insights into the underlying mechanisms, we examined detailed subcellular localization of zRbm14. We imaged the immunostained zebrafish embryos from 3 to 10 hpf (Fig. 1c) at high resolution. Interestingly, we found that zRbm14a mainly distributed in the cytoplasm as numerous puncta at 3 hpf (Fig. 3a). At 6 and 10 hpf, both cytoplasmic and nuclear zRbm14a puncta were observed (Fig. 3a). In addition, a bright perinuclear speckle was often visualized, which was more prominent in the cells at 3 hpf (Fig. 3a, arrowheads).

As antibody to zRbm14b was not available, we microinjected in vitro-transcribed mRNA into one-cell zebrafish embryos to express GFP-zRbm14b. In embryos fixed at approximately 4 hpf, we observed that GFP-zRbm14b also displayed punctate distributions in the cytoplasm and nucleus (Fig. 3b). This also excluded the possibility that the cytoplasmic puncta of zRbm14a were due to non-specific immunostaining of the antibody.

### zRbm14b phase separates in vitro through its IDR

As mammalian Rbm14 is a major component of paraspeckles^6^, we speculated that the cellular puncta of zRbm14 (Fig. 3a, b) could also be RNP compartments assembled through protein phase separation^6,15,52^. We thus investigated whether zRbm14b could undergo phase separation. Using sequence alignment and a PLD detection algorithm^1^, we found that the PLD feature of Rbm14 is not well conserved in evolution. The PLDs of human and mouse Rbm14 cover approximately 300 amino acids and contain 27 conserved “YXXQ” motifs^6^, whereas the *Xenopus* PLD covers approximately 100 residues with 8 “YXXQ” motifs (Fig. 3c; Supplementary Fig. 3a). The C-terminal region of zRbm14b, however, contains only two short PLDs and three “YXXQ” motifs; it displays poor sequence similarity as well to its amphibian and mammalian orthologues as compared to its N-terminal RRM region (Fig. 3c; Supplementary Fig. 3a). Nonetheless, database analysis^53^ suggests that the C-terminus of zRbm14b contains an IDR.

We then expressed and purified from bacteria polyhistidine-tagged (His) GFP or His-GFP-tagged zRbm14b and zRbm14bIDR (Fig. 3d; Supplementary Fig. 3b). We also created zRbm14bIDR18S by mutating 18 Y residues in the putative IDR into S (Fig. 3d; Supplementary Fig. 3b) to hopefully attenuate the phase separation ability^3,6,10^. We found that the bacterially expressed zRbm14b and mutants were not as severely degraded as their human counterparts (Supplementary Fig. 3b)^6^.

As the hydrogel formation of human Rbm14 requires very high protein concentration (~600 μM), long incubation time (≥48 h), and low temperature (4 °C)^6^, we performed liquid droplet formation assays^3^ at physiological temperature (25 °C). GFP-positive droplets were observed with their sizes increasing in a concentration-dependent manner (from 10 to 40 μM) when shifting purified His-GFP-zRbm14bIDR from 0 to 25 °C for 60 min (Supplementary Fig. 3c). Adding polyethylene glycol (PEG8000) as a crowding reagent^3^ to 1% markedly enhanced the liquid droplet formation at 25 °C (Fig. 3e). In the presence of 1% PEG, both His-GFP-tagged zRbm14b and zRbm14bIDR formed liquid droplets at 10 μM upon the incubation for 5 min (Fig. 3f). The droplets were able to rapidly fuse with one another (Fig. 3g), confirming their liquid property. By contrast, neither His-GFP nor His-GFP-zRbm14bIDR18S formed liquid droplets, even at 40-μM concentration (Fig. 3f). Thus, zRbm14b is able to phase separate into liquid droplets through its IDR.

### Phase separation property underlies punctate subcellular distributions and embryonic functions of zRbm14b

**Fig. 4:**
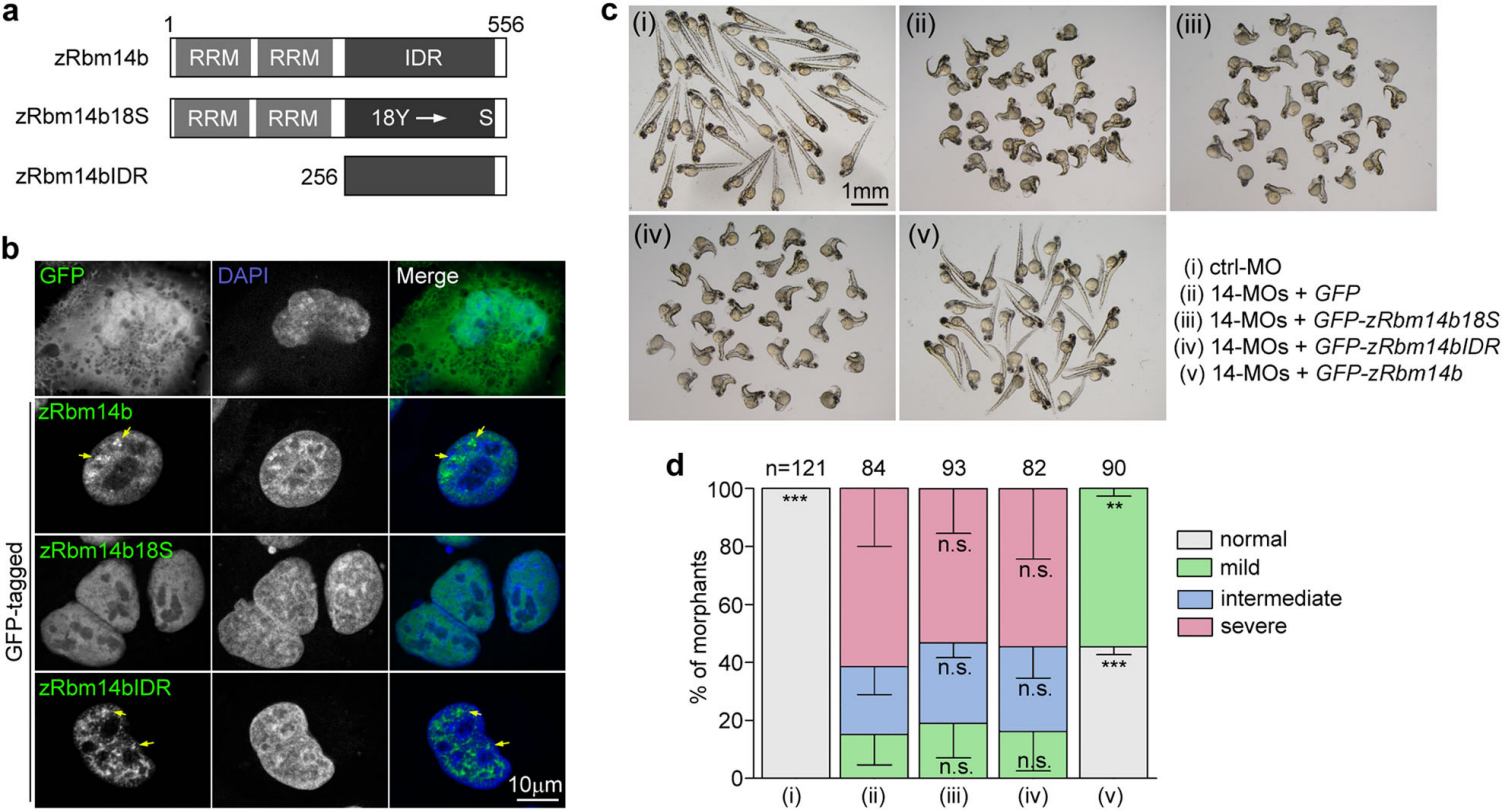
The phase separation property and RRM region of zRbm14b are both essential for proper dorsoventral patterning. **a** Diagrams of zRbm14b and its mutants. Detailed mutation sites in zRbm14b18S are provided in Supplementary Fig. 3a. **b** Subcellular localizations of GFP-tagged zRbm14b, zRbm14b18S, and zRbm14bIDR in HeLa cells. HeLa cells were transfected for 48 h with the intact plasmids for the in vitro mRNA productions (**c**). Nuclear DNA was stained with DAPI. Arrows indicate typical nuclear puncta. **c**, **d** zRbm14b18S and zRbm14bIDR failed to rescue the dorsalized phenotypes of *rbm14* morphants. Zebrafish embryos at the one-cell stage were co-injected with 14-MOs (total 8 ng per embryo) and in vitro-transcribed mRNA (300 pg mRNA per embryo) coding for GFP, GFP-zRbm14b, GFP-zRbm14b18S, or GFP-zRbm14bIDR (also see Supplementary Figs. 1f and 4a). Embryos injected with 8 ng of ctrl-MO served as a negative control. The samples were imaged at 72 hpf (**c**). The quantification results (**d**), based on the criteria and examples in Fig. 1e and represented as mean ± SD, were from three independent experiments. Student’s *t*-test against the *GFP* mRNA-injected populations: n.s., no significance; **P < 0.01; ***P < 0.001. Total number of embryos analyzed are listed over each histogram

To assess whether the phase separation property of zRbm14b is required for the dorsoventral patterning of zebrafish embryos, we constructed plasmids to express GFP-tagged zRbm14b18S and zRbm14bIDR (Fig. 4a), in addition to the one for GFP-zRbm14b (Figs. 1f and 3b; Supplementary Fig. 1f). When expressed in HeLa cells, these proteins predominantly localized in the nucleus (Fig. 4b). Consistent with their phase separation abilities (Fig. 3f), both zRbm14b and zRbm14bIDR showed punctate distribution patterns in the nucleus, whereas zRbm14b18S was evenly dispersed in the nucleoplasm (Fig. 4b). When the mRNAs were in vitro-transcribed from these plasmids and co-injected with 14-MOs to express these proteins in zebrafish embryos (Supplementary Figs. 1f and 4a), GFP-zRbm14b largely reduced the ventralization defects of the *rbm14* morphants as compared to GFP (Fig. 4c, d), as shown previously (Fig. 1f, g). GFP-zRbm14b18S and GFP-zRbm14bIDR, however, were unable to rescue the defects (Fig. 4c, d). Therefore, the phase separation property of zRbm14b is essential for its punctate subcellular distributions and proper dorsoventral patterning. Neither its RRM region (as in zRbm14b18S) nor its IDR alone is sufficient for the embryonic development.

### The IDR of zRbm14b exhibits significant functional interchangeability with other IDRs

We sought to confirm that zRbm14b indeed functions in vivo through phase separation. We reasoned that, if the IDR of zRbm14b was mainly, or even solely, used for phase separation-induced protein network formation in vivo, it could be functionally replaced by the phase separation domains of other protein(s), especially those functioning in the same subcellular compartments and capable of co-phase separation with Rbm14.

We have previously demonstrated that xBuGZΔN, the low complexity region of *Xenopus* BuGZ, is able to phase separate to regulate the assembly of the spindle matrix and spindle microtubules^3^. BuGZ is also a nuclear protein important for pre-mRNA processing in interphase^54^ but its low complexity region is not a PLD. As Rbm14 was identified as a candidate spindle matrix protein by mass spectrometry^55^, we firstly explored whether the IDRs of zRbm14b and xBuGZ could co-phase separate. Indeed, when purified His-GFP-zRbm14bIDR was mixed with His-RFP-xBuGZΔN, both proteins formed well inter-mingled liquid droplets (Supplementary Fig. 4b, c). By contrast, His-GFP-zRbm14bIDR18S was not incorporated into the liquid droplets of His-RFP-xBuGZΔN (Supplementary Fig. 4c). Therefore, we firstly examined a chimeric protein containing the RRM region of zRbm14b and the IDR of xBuGZ, xBuGZΔN.

**Fig. 5:**
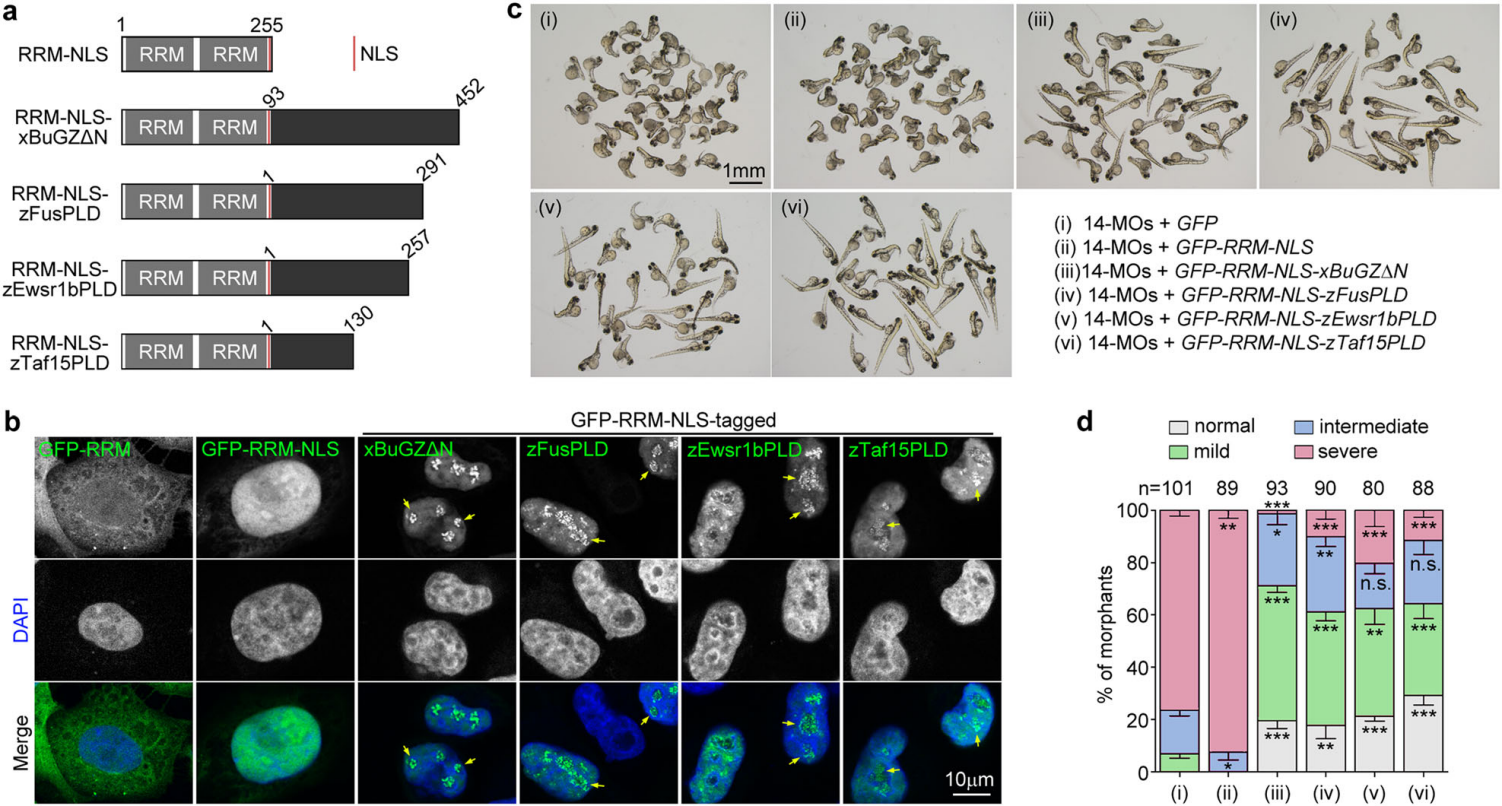
The IDR of zRbm14b can be functionally replaced with other phase separation domains in vivo. **a** Diagrams of chimeric proteins examined. RRM, the RRM region of zRbm14b; NLS, the nuclear localization signal of large T antigen; xBuGZΔN, the IDR of xBuGZ; zFusPLD, zEwsr1bPLD, or zTaf15PLD, the PLD domain of zebrafish Fus, Ewsr1b, or Taf15 (also see Supplementary Fig. 4e). Numbers indicate amino acid positions in each intact protein. **b** Subcellular localization of the indicated GFP-tagged proteins. HeLa cells were transfected with the intact plasmids used for in vitro mRNA productions (**c**) for 48 h and fixed with 4% paraformaldehyde. Nuclear DNA was stained with DAPI. Arrows indicate representative nuclear foci. **c**, **d** The phase separation domains of xBuGZ, zFus, zEwsr1b, and zTaf15 were partially redundant to the IDR of zRbm14b. Zebrafish embryos at the one-cell stage were co-injected with 14-MOs (8 ng per embryo) and the indicated in vitro-transcribed mRNA (300 pg mRNA per embryo) (also see Supplementary Fig. 4d, f) and imaged at 72 hpf (**c**). The quantification results (**d**), based on the criteria and examples in Fig. 1e and represented as mean ± SD, were from three independent experiments. Student’s *t*-test against the *GFP* mRNA-injected populations: n.s., no significance; *P < 0.05; **P < 0.01; ***P < 0.001. Total numbers of embryos analyzed are listed over the histogram

As the RRM region of zRbm14b and xBuGZΔN lacked a nuclear localization signal (NLS) (Fig. 5a, b; Supplementary Fig. 3a)^3^, we fused the NLS of large T antigen^56^ to the C-terminus of the RRM region so that both GFP-RRM-NLS and GFP-RRM-NLS-xBuGZΔN were targeted to the nucleus as confirmed in HeLa cells (Fig. 5a, b). In contrast to the relatively homogeneous distributions of GFP-RRM-NLS, however, GFP-RRM-NLS-xBuGZΔN formed bright nuclear foci (Fig. 5b), suggestive of its phase separation in the cells. More importantly, expression of GFP-RRM-NLS-xBuGZΔN, but not GFP or GFP-RRM-NLS, in zebrafish embryos significantly reduced the ventralization defects of the *rbm14* morphants (Fig. 5c, d; Supplementary Fig. 4d).

Many hnRNP proteins including EWSR1 and Taf15 have been shown to co-phase separate with FUS in vitro through their PLD^10^. As FUS, EWSR1, and Taf15 are also paraspeckle components^6,32,33^, we speculated that Rbm14, which is structurally similar to hnRNP proteins, could complex with them in vivo through phase separation. We thus investigated whether the PLDs of zebrafish Fus, Ewsr1, and Taf15 (Supplementary Fig. 4e) might also be able to functionally replace the IDR of zRbm14b. Similar to GFP-RRM-NLS-xBuGZΔN, GFP-RRM-NLS-tagged zFusPLD, zEwsr1bPLD, and zTaf15PLD also displayed bright nuclear foci in HeLa cells (Fig. 5a, b). Furthermore, they also significantly rescued the ventralization defects of the *rbm14* morphants (Fig. 5c, d; Supplementary Fig. 4f). Together, these results demonstrate that the phase separation property mediated by IDRs of different proteins exhibits substantial functional interchangeability in vivo.

### Cellular zRbm14b phase separates into RNA granules

**Fig. 6:**
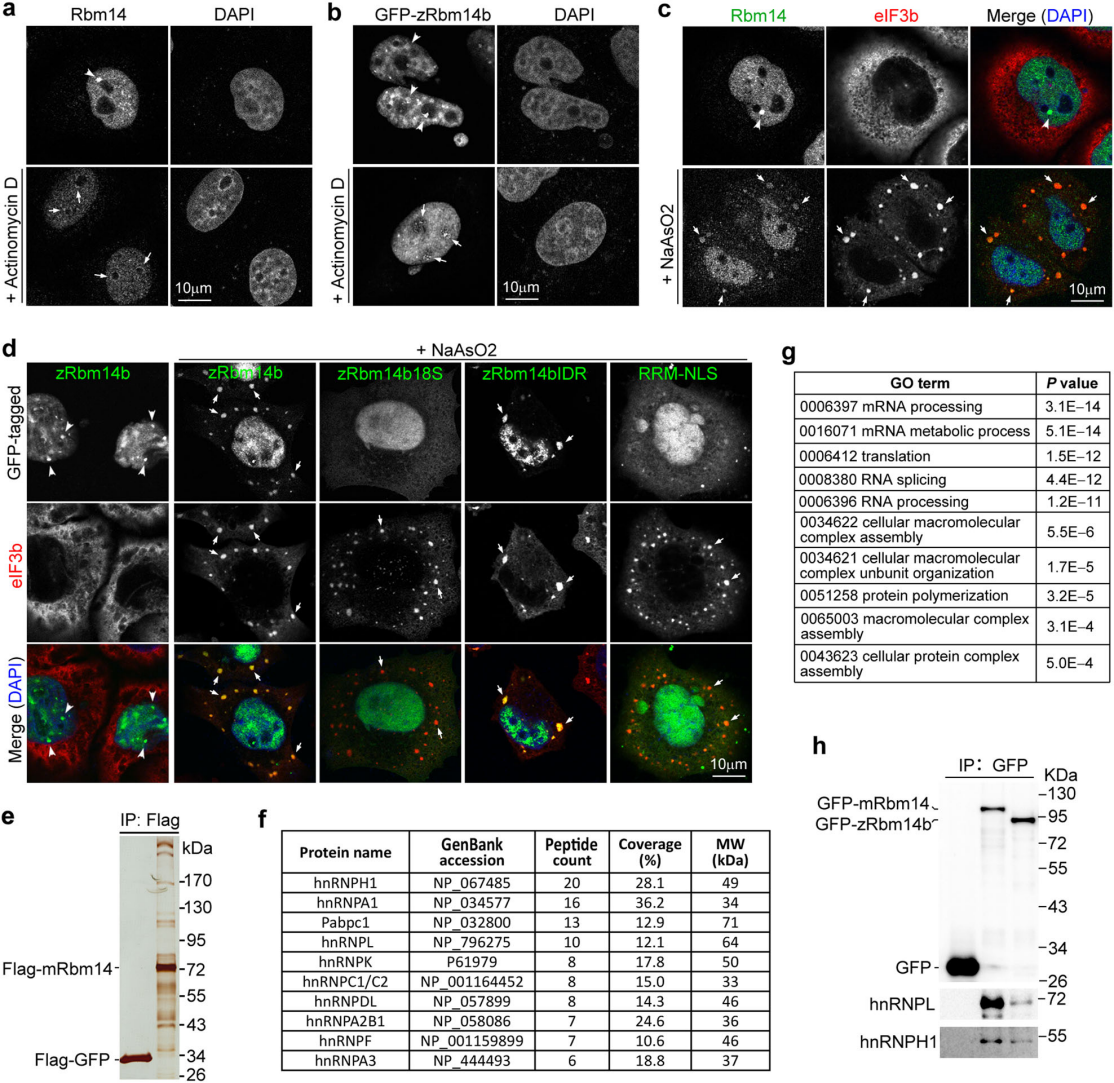
Rbm14 partitions into RNP compartments and complex with RNA-binding proteins important for RNA metabolism. **a**, **b** The nucleoplasmic puncta (arrowheads) of endogenous human Rbm14 and exogenous zRbm14b were sensitive to RNA pol II activity. HeLa cells that were untransfected (**a**) or transiently transfected to express GFP-zRbm14b (**b**) were treated with actinomycin D for 60 min prior to fixation. Nuclear DNA was visualized with DAPI. Arrows indicate fluorescent signals at nucleolar caps. **c** Human Rbm14 localized to stress granules (SGs; arrows). HeLa cells were treated with sodium arsenite to induce SGs. Untreated cells were used as control. eIF3b served as an SG marker. **d** zRbm14b translocated into SGs (arrows) through phase separation. HeLa cells transiently expressing the indicated zRbm14b mutants were treated with sodium arsenite. **e** Mouse Rbm14 associated with large protein complexes. Flag-GFP and Flag-mRbm14 expressed in mouse embryonic stem cells were immunoprecipitated using anti-Flag resin. The immunoprecipitates were resolved by SDS-PAGE and silver stained. **f** The top 10 hits of potential Rbm14-associated proteins. The immunoprecipitates of Flag-GFP and Flag-Rbm14 were subjected to shotgun mass spectrometric analysis. The top ten hits identified exclusively from the latter sample, according to total peptide count, are listed. **g** Top ten gene ontology (GO) term hits on candidate Rbm14-associated proteins. Only proteins identified exclusively in the Flag-mRbm14 sample were used for the analysis. **h** zRbm14b also associated with hnRNP proteins. GFP-tagged mRbm14 and zRbm14b expressed in HeLa cells were immunoprecipitated using anti-GFP resin and immunoblotted to detect the indicated proteins

Inhibition of RNA polymerase II (pol II) activity with drugs such as actinomycin D is known to abolish mRNA-containing RNA granules including paraspeckles. As a result, many RNA-binding proteins such as Fus and Taf15, which also show paraspeckle localization, become enriched in perinucleolar caps^34,57–61^. To understand the molecular functions of zRbm14, we investigated whether its subcellular puncta (Figs. 3a, b and 4b) were mRNA-containing granules. When HeLa cells were treated with actinomycin D, the nucleoplasmic puncta of both endogenous Rbm14 and GFP-zRbm14b disappeared; both proteins became accumulated at the nucleolar regions (Fig. 6a, b). Therefore, zRbm14b is capable of partitioning into RNA granules related to pol II-mediated transcriptions.

Many paraspeckle components have been shown to translocate into SGs, which are stress-induced, evolutionarily conserved cytoplasmic condensates abundant in translationally arrested mRNAs, translation initiation factor (eIF) components, and a large variety of RNA-binding proteins^13,14,62,63^. We thus induced oxidative stress in HeLa cells with sodium arsenite^14,62^ and found that endogenous Rbm14 translocated into cytoplasm foci positive for the SG marker eIF3b (Fig. 6c)^64,65^. GFP-tagged zRbm14b and zRbm14bIDR also potently translocated into SGs when expressed in HeLa cells, whereas zRbm14b18S and RRM-NLS were absent from SGs (Fig. 6d). These results further support functional conservation of Rbm14 from fish to mammals.

### Rbm14 complexes with other RNA-binding proteins

For clues on Rbm14-associated proteins in the RNP compartments, we respectively expressed Flag-mRbm14 and Flag-GFP in mouse embryonic stem cells and performed co-immunoprecipitation. Silver staining indicated association of many proteins with Flag-mRbm14 in mouse embryonic stem cells as compared to Flag-GFP (Fig. 6e). Shotgun mass spectrometry identified 157 potential Rbm14-associated proteins. The top ten hits, including nine hnRNP family proteins and one poly(A)-binding protein (Pabpc1) (Fig. 6f), were all RNA-binding proteins involved in translation, transcription, alternative splicing, and mRNA stability^12,66^. Many of these proteins are also reported to localize to paraspeckles (hnRNPH1, -A1, -L1, -K, and -F)^67^ and/or SG (hnRNPA3, -A1, -A2B1, -K, and Pabpc1)^14,26,68^, and undergo in vitro phase separation (hnRNPH1, -A1, -DL, -A2B1, and -A3)^10^. GO enrichment analysis of the interactome showed that the top five hits were related to RNA processing, metabolic process, splicing, and translation, followed by the next five hits related to cellular macromolecular complex formation (Fig. 6g). Nanog, Smads, or Id proteins, however, were not hit by the mass spectrometry.

We then expressed GFP-tagged mRbm14 or zRbm14b in HeLa cells and performed immunoprecipitation using anti-GFP resin. Immunoblotting with available antibodies indicated that hnRNPL and -H1 associated with both GFP-mRbm14 and -zRbm14b, but not GFP (Fig. 6h). Together with previous reports^27,28,31^, these results suggest that both mammalian and zebrafish Rbm14 proteins form complexes with other RNA-binding proteins to regulate RNA metabolism.

### Zebrafish *rbm14* morphants display large-scale gene downregulations and increased alternative splicing

To understand whether or not zRbm14 functions as a regulator specific to the BMP-related genes, we conducted transcriptomic deep sequencing on control and *rbm14* morphants at 10 and 24 hpf, respectively. At each time point, total RNAs were prepared from 50 ctrl-MO-injected and 50 14-MOs-injected embryos, respectively. The samples were validated by culturing the same batch of remaining embryos to 72 hpf (Supplementary Fig. 5a, b). Furthermore, the deep sequencing results confirmed severe intron retention between exons 4 and 5 of *rbm14b* in the *rbm14* morphants (Supplementary Fig. 5c), caused by 14b-MO that was designed to block the splicing of the intron (Supplementary Fig. 1a).

**Fig. 7:**
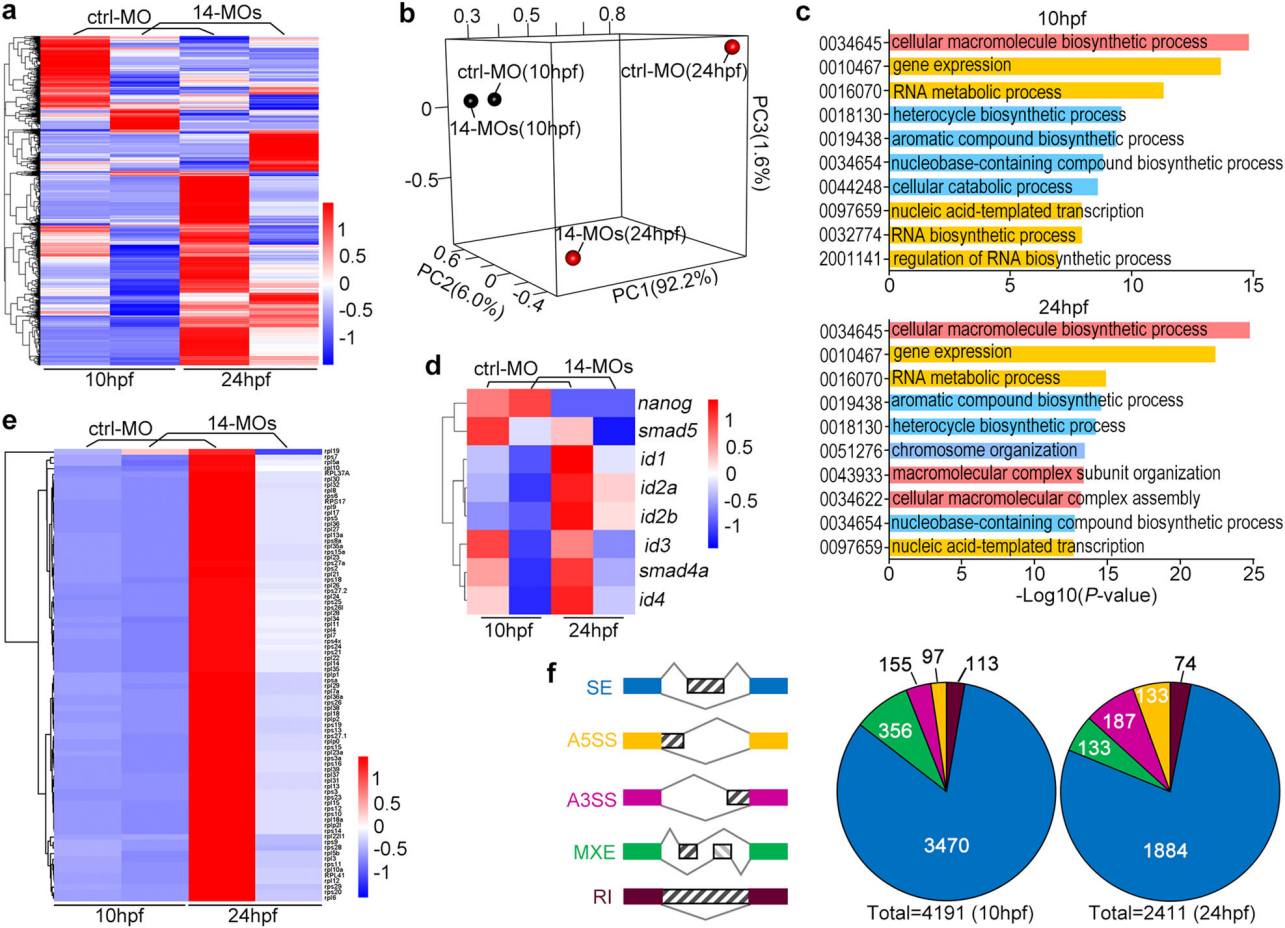
Zebrafish *rbm14* morphants display transcriptomic downregulation and alternative splicing. **a** A heat map of differentially expressed genes in control (ctrl-MO) and *rbm14* (14-MOs) morphants at 10 or 24 hpf. **b** The principal component analysis to show variance in the gene expression profiles of the control and *rbm14* morphants. We found that the first principal component (PC1) explains 92.2% of the variance, while PC2 and PC3 explain 6.0% and 1.6%, respectively. **c** The top 10 GO term events of the differentially expressed genes. **d** A heat map of *smads*, *ids*, and *nanog* transcripts. **e** A heat map of transcripts of 81 ribosomal protein genes. In cases when multiple Ensembls corresponded to the same gene, only the one with the highest transcript levels was used for analysis. **f** Analysis of differential AS events in the *rbm14* morphants. SE, skipped exon; A3SS, alternative 3′ splicing site; A5SS, alternative 5′ splicing site; MXE, mutually exclusive exon; RI, retained intron

We analyzed 22,053 transcripts with TPM >1 in at least one sample. Compared to the control morphants, the majority of differentially expressed genes in the *rbm14* morphants were downregulated. At 10 hpf, 5631 transcripts were altered by more than twofold; 84% of them were downregulated in the *rbm14* morphants (Fig. 7a). At 24 hpf, 6279 transcripts were altered by more than twofold and 68% of them were downregulated (Fig. 7a). Principal components analysis (PCA) revealed that the control and *rbm14* morphants were more closely related at 10 hpf than at 24 hpf (Fig. 7b), indicating that the difference in their gene expression profiles increased over time. GO enrichment analysis showed that the altered transcripts were associated with more than 360 categories of biological functions above the threshold (*p* < 0.05). The top 10 events were mainly related to mRNA levels and cellular macromolecular complexes (Fig. 7c).

We found that *smad4a*, *smad5*, and multiple *ids* were among genes downregulated at both 10 and 24 hpf (Fig. 7d), consistent with our previous analyses (Figs. 1 and 2). Only *nanog* was slightly upregulated at 10 hpf in the *rbm14* morphants and expressed at low levels at 24 hpf in both control and *rbm14* morphants (Fig. 7d). As *nanog* mRNA levels are only robust before 6 hpf and decline dramatically afterwards^69^, such deep sequencing results at 10 and 24 hpf might not reflect situations when the embryos were undergoing the dorsoventral patterning. Notably, the transcripts of all ribosomal proteins were downregulated by 87% to 57% (mean = 68%) at 24 hpf (Fig. 7e), which could attenuate ribosome biogenesis and consequently global protein syntheses. At 10 hpf, most transcripts were mildly downregulated. The average downregulation was by 19% for all the transcripts (Fig. 7e).

In the *rbm14* morphant samples, 4191 differential alternative splicing events were identified at 10 hpf, the majority of which (83%) belonged to skipped exon (SE) (Fig. 7f)^70^. There were 2411 differential alternative splicing events at 24 hpf, also with preference to SE (78%) (Fig. 7f). These results suggest a general regulatory role of zebrafish Rbm14 in expressions and mRNA processing of a large pool of genes.

## Discussion

We found that Rbm14 is a critical ventralization factor (Fig. 1). The two zebrafish paralogues, *rbm14a* and *rbm14b*, are maternally expressed genes that function redundantly in the dorsoventral patterning during gastrulation (Figs. 1 and 2; Supplementary Fig. 1d). Our results suggest that zRbm14 functions through phase separation to regulate multiple aspects of RNA metabolism (Figs. 3–7). Furthermore, mammalian and zebrafish Rbm14 orthologues share similar properties and functions (Figs. 2, 6 and 7), despite their poor sequence homologies outside the RNA-binding regions (Supplementary Fig. 3a). For simplicity, we do not distinguish species in the following discussions unless necessary.

We demonstrated that phase separation is essential to the functions of Rbm14 in vivo. During evolution, the phase separation ability, but not the primary sequence, is conserved in the IDR of Rbm14 (Fig. 3; Supplementary Fig. 3a)^6^. Mutational analysis on zRbm14b’s IDR (Figs. 3 and 4) as well as its significant functional compensation by those of other proteins (Fig. 5; Supplementary Fig. 4) provided solid evidence that its major role is phase separation. Furthermore, such results suggest that the phase separation roles of IDRs are less context-specific and can be interchangeable. Although extensive point mutations are commonly used in the field to impair protein phase separation ability, a strong concern against the use of such mutants in physiological assays is that some mutations may affect other functions irrelevant to phase separation. Therefore, similar chimeric proteins could be used as an approach to confirming in vivo physiological significance of protein phase separation, though the replacements may be less efficient than the original IDR as in the case of zRbm14b (Fig. 1f, g, 4c, d vs. Fig. 5c, d). In this study we selected IDRs from potential Rbm14 partners. Whether a randomly picked IDR will do or to what extent IDRs are functionally interchangeable proteome-wide, however, remains to be clarified in the future.

Rbm14 appears to function in different RNP compartments in a context-dependent manner to regulate RNA metabolism, such as RNA transcription, alternative splicing, storage, stabilization, and translation. Rbm14 is initially identified as a transcription co-activator capable of affecting alternative splicing^27,28^. Our analyses on both the interactome (Fig. 6e–h) and the differential transcriptome (Fig. 7) also suggest involvement of Rbm14 in gene expression, transcription, and metabolic process with other RNA-binding proteins. The large-scale downregulation and alternative splicing of genes in *rbm14* morphants (Fig. 7; Supplementary Table 1) further strengthen the importance of Rbm14. In addition to the known paraspeckle localization of mammalian Rbm14 (Fig. 6a)^6,33^, we found that it also translocated into cytoplasmic SGs under oxidative stress (Fig. 6c). The localizations of zRbm14b in nuclear RNP compartments and cytoplasmic SGs when exogenously expressed in HeLa cells (Figs. 3b and 6b, d) allow us to conclude that the cytoplasmic and nuclear puncta of zRbm14 in zebrafish embryonic cells (Fig. 3a, b) are RNA granules. Therefore, even if paraspeckles are not present in zebrafish because *Neat1* is mammal-specific^34^, other nuclear RNP compartments that recruit zRbm14 still exist. Whether the cytoplasmic zRbm14 puncta (Fig. 3a, b) are SGs formed in response to various stresses^13^, such as the redox stress^71^, remains to be clarified. In addition, cells are also known to contain other cytoplasmic RNA granules, e.g., the processing bodies (P bodies), that regulate RNA metabolism such as RNA storage and translation regulation^52,72–74^.

Our results suggest that Rbm14 functions in ventralization by sustaining the levels of other important ventralization factors such as Nanog, Smad4, and Smad5 (Fig. 2). Although thousands of mRNAs (and likely their coding proteins) in the *rbm14* morphants were affected (Fig. 7), the embryos manifested relatively defined phenotypes (Fig. 1). Therefore, it appears that developing early embryos are more sensitive to the levels of certain key regulators. During vertebrate gastrulation, an extracellular BMP gradient causes increased activations of BMP pathway transcription factors such as the Smad4–Smad5 heterodimer formation along the dorsoventral axis to induce ventral cell fate^44–46^. Nanog contributes to the specific activation of the BMP signaling in ventrolateral endoderm to fine tune the complicated ventralization process^51^. In Rbm14-depleted P19 cells, Smad4, Smad5, and Nanog were markedly reduced (Fig. 2a, b, g). The attenuated BMP effectors Id1 and Id2 further indicate impaired BMP signaling (Fig. 2a, b)^49,50^. In zebrafish embryos, the expression regions of zRbm14a (Fig. 1c) overlap with those of *smad4* (ref. ^75^), *smad5* (refs. ^76–78^), and *nanog*^69^. Although detailed molecular mechanisms are still unclear, the reduced levels of these ventralization factors (Fig. 2) could be attributed to downregulations of their mRNAs and other proteins important for translation (Fig. 7d, e). Loss of Rbm14 from its RNP compartments (Figs. 3a, b and 6a–d)^6^ could also hinder the accessibility of the mRNAs to the translation machinery, resulting in reduced protein levels without affecting mRNA abundance.

## Materials and methods

### Plasmids, siRNAs, and antibodies

For antibody production, the full-length zebrafish *rbm14a* cDNA (GenBank accession NM_001115144) was amplified by RT-PCR and cloned into pGEX-4T-1 between the *Bam*HI and *Not*I sites to express GST-zRbm14a.

For rescue experiments in zebrafish, the full-length *EGFP* cDNA was amplified by PCR from pEGFP-C1 and cloned into the *Bam*HI and *Eco*RI sites of pCS2 to generate pCS2-GFP. The cDNAs coding for the full-length zRbm14b (NM_212808) and mutants, zSmad4 (NM_001122700), zSmad5 (NM_131368), zId1 (NM_131245), zId2b (NM_199541), mNanog (NM_028016.3), xBuGZΔN^3^, and the PLDs of zFus (NM_201083; nucleotides 1–873 starting from the initiation codon), zEwsr1b (NM_212630; nucleotides 1–771), and zTaf15 (NM_001079973; nucleotides 1–390) were amplified by RT-PCR. The *rbm14b18S* cDNA was synthesized by Biosune Biotechnology Shanghai Co.; its codons contain the following 18 Y-to-S mutations compared to the wild-type cDNA: Y260S, Y269S, Y290S, Y298S, Y316S, Y321S, Y350S, Y357S, Y363S, Y382S, Y425S, Y454S, Y455S, Y458S, Y459S, Y462S, Y468S, and Y471S (Supplementary Fig. 3a). These cDNAs were cloned into pCS2-GFP.

To express His-GFP or His-GFP-tagged zRbm14b or mutants in *Escherichia coli*, the *EGFP* cDNA was amplified from pEGFP-C1 by PCR and cloned into pET30a between the *Bgl*II and *Bam*HI sites to form pET30a-GFP. The cDNAs coding for zRbm14b, zRbm14bIDR (amino acids 256–556 of zRbm14b), or zRbm14bIDR18S were amplified by PCR and inserted between the *Bam*HI and *Not*I sites of pET30a-GFP. A sequence coding for a flexible amino acid linker (3×GGGGS) was placed at the 5′ of the *rbm14b* cDNA or mutants during PCR to increase the flexibility of the fused proteins^3^. To express His-RFP-xBuGZΔN in *E. coli*, the cDNAs coding for RFP and xBuGZΔN^3^ were PCR-amplified and inserted in-frame into pET28a.

To express Flag-tagged mRbm14 (NM_019869.3) for co-immunoprecipitation, the full-length cDNA was PCR-amplified to contain a Flag-coding sequence and cloned into pFUGW. A Flag-coding sequence was inserted into pEGFP-C1 to express Flag-GFP. To express GFP-tagged mRbm14, the full-length cDNA was PCR-amplified and cloned into pEGFP-C1 at the *Bgl*II site. All the plasmids used were subjected to sequencing confirmation. Detailed information, including the sequences of PCR primers, is listed in Supplementary Table 1.

siRNAs targeting *mRbm14* were synthesized by Gima Biol Engineering Inc. (Shanghai, China). Control siRNA from Gima was used as a negative control. Their sequences are listed in Supplementary Table 1.

Rabbit polyclonal antibody to zRbm14a was generated by Immune Biotech using purified GST-zRbm14a as antigen and affinity purified using His-zRbm14a. Antibodies used and their dilutions are listed in Supplementary Table 1.

### Cell culture, transfection, and drug treatment

HeLa and mouse embryonic carcinoma P19 cells were cultured in Dulbecco’s modified Eagle medium (DMEM) and DMEM/F12 (Invitrogen), respectively, both supplemented with 10% fetal bovine serum (Biochrom, Cambridge, UK), 2 mM l-glutamine (Sigma), 100 U/ml penicillin (Invitrogen), and 100 U/ml streptomycin (Invitrogen) at 37 °C in an atmosphere containing 5% CO_2_. Mouse embryonic stem cells E14.Tg2a (feeder-free) were maintained on 0.1% gelatin-coated dishes in Glasgow Minimum Essential Medium (GMEM) (Gibco) supplemented with 15% fetal bovine serum, GlutaMAXTM-I (100× stock; Gibco), MEM nonessential amino acids (100× stock; Gibco), 2-mercaptoethanol (1000× stock; Gibco), and 1000 U/ml leukocyte inhibitory factor (Millipore). Transfections were performed by using Lipofectamine RNAiMAX (Life Technologies) for siRNAs or Lipofectamine 3000 (Life Technologies) for plasmids. The cells were transfected for 48 h before being treated for subsequent experiments. To induce SGs, HeLa cells were treated with 1 mM NaAsO_2_ (Innochem) for 60 min at 37 °C and washed with phosphate-buffered saline (PBS) for three times rapidly prior to fixation with paraformaldehyde^14,62^.

### In vitro transcription

In vitro transcription was performed using linearized plasmids and mMESSAGE mMACHINE Kit (Ambion, AM1340). Transcribed mRNAs were purified using MEGAclear™ Purification Kit (Ambion, AM1908), and dissolved in RNase-free water. mRNA concentrations were quantified by using a NanoDrop 2000 spectrophotometers (Thermofisher). Restriction enzymes used to linearize the plasmids and RNA polymerases used for mRNA syntheses are listed in Supplementary Table 1.

### Zebrafish and microinjection

Zebrafish embryos were cultured in Holtfreter’s solution at 28.5 °C and staged as described^37^. MOs (Gene Tools; sequences are listed in Supplementary Table 1) were dissolved in nuclease-free water and injected at 2 nl (containing 4–16 ng MO) per embryo at the one-cell stage using a Narishige IM300 micro-injector. One hundred picograms of GFP mRNA, in vitro-transcribed from pCS2-GFP, were co-injected as injection marker. In rescue experiments, 2 nl of MO solution containing 100–300 pg of in vitro-transcribed mRNA were injected. GFP-positive embryos were collected at 10 hpf for further investigation. Embryos were photographed under an Olympus SZX16 stereo microscope with a SPOT Insight digital camera.

Experiments on zebrafish embryos were performed in accordance with protocols approved by the Institutional Animal Care and Use Committee of Institute of Biochemistry and Cell Biology.

### In situ hybridization

Plasmids harboring the cDNAs for zebrafish *eve1* (ref. ^43^) and *gsc*^42^ were linearized as indicated in Supplementary Table 1. Digoxigenin-UTP-labeled antisense RNA probes were generated by in vitro transcription using DIG RNA Labeling Kit (Roche 11175025910). Whole-mount in situ hybridization of zebrafish embryos was carried out as previously described^79,80^. The embryos were then immersed in glycerol and photographed under a stereo microscope.

### Fluorescent microscopy

Cultured cells grown on coverslips were fixed with 4% fresh paraformaldehyde in PBS for 15 min at room temperature. Fixed cells were permeabilized with 0.5% Triton X-100 in PBS for 15 min and blocked with 4% bovine serum albumin in PBS for 1 h. They were incubated with primary antibodies at 4 °C overnight, followed by three times of wash with 0.5% Triton X-100 in PBS. After incubation with secondary antibodies at room temperature for 1 h, the cells were washed three times and counter-stained with DAPI (1 μg/ml; Sigma-Aldrich) for 15 min. After three times of wash, the coverslips were mounted onto glass slides using fluorescent mounting medium (Dako). Images was performed with a Leica TCS SP8 confocal microscope.

Zebrafish embryos were manually dechorionated and fixed in 4% PFA in PBS at 4 °C overnight. After washing with PBS, embryos were sequentially dehydrated at room temperature in 25%, 50%, 75%, and 100% methanol/PBS, 5 min each, and incubated in 100% methanol overnight at −20 °C. The embryos were rehydrated in methanol/PBS (75%, 50%, and 25%, 5 min each) at room temperature, washed three times with 0.1% Tween-20 in PBS, followed by incubation in blocking buffer (2% bovine serum albumin, 0.5% goat serum, 1% DMSO, 0.5% Trion X-100 in PBS) for 1 h at room temperature. The embryos were then incubated with anti-zRbm14a antibody diluted in the blocking buffer overnight at 4 °C. After three times of wash with 0.1% Tween-20 in PBS, Alexa Fluor 488-conjugated secondary antibody was added and incubated overnight at 4 °C. After three times of wash, nuclear DNA was stained with DAPI (2 μg/ml) for 30 min. The embryos were washed for three times and photographed with an Axio Zoom V16 microscope (Zeiss). High-resolution images were acquired with a Leica TCS SP8 confocal microscope as single optical sections.

### RT-PCR analyses

Total RNAs were extracted from 50 zebrafish embryos at 0 or 10 hpf using TRI Reagent (Sigma-Aldrich). mRNAs were reverse-transcribed into cDNAs using oligo dT and SuperScript™ III Reverse Transcriptase (Invitrogen).

Primer pairs used for real-time quantitative PCR (qPCR) analyses were designed by using the Peal-Primer software (http://perlprimer.sourceforge.net). Their sequences are listed in Supplementary Table 1. Real-time PCR was performed using an ABI 7500-Fast system and the power SYBR Green PCR master mix (ABI 4367659). PCR mixtures were incubated at 95 °C for 5 min, followed by 30 s at 95 °C, 20 s at 60 °C, and 30 s at 72 °C for 40 cycles and a final 10-min incubation at 72 °C. Relative expression levels were normalized against the internal control β-actin.

To analyze MO efficiency, cDNAs of *rbm14a* and *rbm14b* were amplified by denaturation at 95 °C for 5 min, followed by 30 s at 95 °C, 30 s at 58 °C, and 30 s at 72 °C for 30 cycles and a final 10-min incubation at 72 °C, using the primers for qPCR.

### Protein purification

Proteins were expressed in the *E. coli* BL21-CondonPlus (DE3) strain after 1 mM IPTG induction for 12–16 h at 16 °C. To purify GST-zRbm14a, 25 ml of cold PBS containing 1% Triton X-100, 3 mM DTT, 1 mM PMSF, and 1 mg/ml protease inhibitor cocktail (Calbiochem) were used to re-suspend bacteria pellets from each liter of bacterial culture. Following a 30-min incubation on ice, the bacteria were lysed in an Ultra-high Pressure Cell Disrupter (JNBIO, JN-02C). The bacterial lysates from 1-liter culture were incubated with 2.5 ml of 50% glutathione agarose (Sigma-Aldrich) and loaded into a column. The column was washed with 200 ml of the buffer and 200 ml of the buffer without DTT. The bound protein was eluted using the buffer containing 250–500 mM reduced glutathione and concentrated to 1–20 mg/ml in PBS using Amicon Ultra Centrifugal Filters (Millipore) depending on the protein. Bacteria expressing His-tagged proteins were re-suspended in cold NTA buffer (50 mM NaH_2_PO_4_, 500 mM NaCl, 10% glycerol, and 10 mM Imidazole, pH 8.0) containing 1 mM PMSF, 1 mg/ml protease inhibitor cocktail. The bacterial lysates were incubated with Ni-NTA resin (Qiagen) at 4 °C for 1 h. The bound proteins were eluted using 500 mM imidazole in NTA buffer and concentrated to 1–20 mg/ml in PBS. Purified proteins were aliquoted, snap frozen in liquid nitrogen, and stored at −80 °C.

### Phase separation assay

Purified proteins were thawed on ice and diluted into ice-cold PBS buffer with or without 1% PEG8000, followed by incubation at 25 °C for 5 min or more. Five microliters of the protein solution were loaded into a flow chamber, consisting of a coverslip on top of a glass slide, separated slightly with two pieces of double-sided adhesive tape. Samples were imaged immediately using an Olympus BX51 fluorescence microscope. Droplet fusion events were observed by imaging at 0.5-s intervals.

### mRNA deep sequencing and data analysis

Total RNA of each sample was extracted from 50 pooled zebrafish embryos using TRIzol Reagent (Invitrogen). Total RNA of each sample was quantified and qualified by an Agilent 2100 Bioanalyzer (Agilent Technologies), NanoDrop (ThermoFisher Scientific), and 1% agarose gel. One microgram total RNAs with RIN (RNA integrity number) value above 7 were used for library preparation. Next-generation sequencing library preparations were constructed according to the manufacturer’s protocol (NEBNext® UltraTM RNA Library Prep Kit for Illumina®). The poly(A) mRNA isolation was performed using NEBNext Poly(A) mRNA Magnetic Isolation Module (NEB). The mRNA fragmentation and priming were performed using NEBNext First Strand Synthesis Reaction Buffer and NEBNext Random Primers. First-strand cDNAs were synthesized using ProtoScript II Reverse Transcriptase and the second-strand cDNAs were synthesized using Second Strand Synthesis Enzyme Mix. The double-stranded cDNAs, purified by AxyPrep Mag PCR Clean-up (Axygen), were then treated with End Prep Enzyme Mix to repair both ends and add a dA-tailing in one reaction, followed by a T-A ligation to add adaptors to both ends. Size selection of the Adaptor-ligated DNAs was then performed using AxyPrep Mag PCR Clean-up (Axygen), and fragments of ~360 bp (with the approximate insert size of 300 bp) were recovered. Each sample was then amplified by PCR for 11 cycles using P5 and P7 primers, with both primers carrying sequences that would anneal with sites on the flow cell to perform bridge PCR and P7 primer carrying a six-base index allowing for multiplexing. The PCR products were cleaned up using AxyPrep Mag PCR Clean-up (Axygen), validated using an Agilent 2100 Bioanalyzer (Agilent Technologies), and quantified by a Qubit 2.0 Fluorometer (Invitrogen). Then the libraries with different indices were multiplexed and loaded on an Illumina HiSeq instrument according to the manufacturer’s instructions (Illumina). Sequencing was carried out using a 2×150-bp paired-end (PE) configuration. Image analysis and base calling were conducted by the HiSeq Control Software (HCS) + OLB + GAPipeline-1.6 (Illumina) on the HiSeq instrument. The pass filter data of FASTQ format were processed by Trimmomatic (v0.30) to remove technical and low-quality sequences.

Reference genome sequences and gene model annotation files of relative species were downloaded from ENSEMBL (Danio_rerio.GRCz10.86). Hisat2 (v2.0.1) was used to index reference genome sequence^81^. Clean data were then aligned to reference genome via software Hisat2 (v2.0.1). Transcripts in the FASTA format were converted from known gff annotation file and indexed properly. Then, with the file as a reference gene file, HTSeq (v0.6.1)^82^ was used to estimate gene and isoform expression levels from the pair-end clean data.

Only transcripts with TPM >1 in at least one sample were used for differential gene expression analysis. *tmem50a* was used as the reference gene for gene expression level normalization^83^. Only transcripts with absolute fold change >2 between samples were considered to be differential. Gene Ontology (GO) enrichment analysis of differentially expressed genes was done through the Database of Annotation, Visualization and Integrated Discovery (DAVID)^84^. GO terms with *P* value <0.05 were considered significantly enriched by differential expressed genes^85^. For identifying differential AS events across samples, rMATS (4.0.2)^86^ were used with –*c* = 0.00001 parameter. The differential splicing events were calculated at the threshold FDR <0.05 and |Δ*ψ*| ≥ 5%.

### Statistical analysis

Two-tailed unpaired student’s *t*-test was performed to calculate *P* values using GraphPad Prism version 5.0 (GraphPad Software, San Diego). Differences were considered significant when *P* < 0.05. Only results from three independent experiments were subjected to the *t*-test.

## Supplementary information

Supplementary information

Supplementary information

## Data Availability

The accession number for the deep sequencing data is GSE128984. Other data supporting the reported results are available upon request to X.Z.

## Supplementary information

**Supplementary Information** accompanies the paper at (10.1038/s41421-019-0106-x).
