# Supplementary material for: Regulation of zebrafish dorsoventral patterning by phase separation of RNA-binding protein Rbm14
Source: Cell Discov. 2019 Jul 23;5:37. doi: 10.1038/s41421-019-0106-x (PMC6796953; doi:10.1038/s41421-019-0106-x)
Supplement: Supplementary file 1 — Supplementary information [file 41421_2019_106_MOESM1_ESM.pdf]

Supplementary information

Regulation of zebrafish dorsoventral patterning by phase separation of RNA-binding protein Rbm14

Yue Xiao[1], Jiehui Chen[1], Yihan Wan[1,2], Qi Gao[1], Naihe Jing[1], Yixian Zheng[2,*], and Xueliang Zhu[1,*]

[1]. State Key Laboratory of Cell Biology, CAS Center for Excellence in Molecular Cell Science, Shanghai Institute of Biochemistry and Cell Biology, Chinese Academy of Sciences; University of Chinese Academy of Sciences, 320 Yueyang Road, Shanghai 200031, China

[2]. Department of Embryology, Carnegie Institution for Science, 3520 San Martin Dr., Baltimore, MD 21218, USA

*. Correspondence: Yixian Zheng (zheng@ciwemb.edu) and Xueliang Zhu (xlzhu@sibcb.ac.cn)

Leading contact: Xueliang Zhu

Email: xlzhu@sibcb.ac.cn

Phone: 86-21-54921406

Mailing address: Shanghai Institute of Biochemistry and Cell Biology, 320 Yueyang Road, Shanghai 200031, China

The supplementary information contains 5 supplementary figures, their legends, and 3 references.

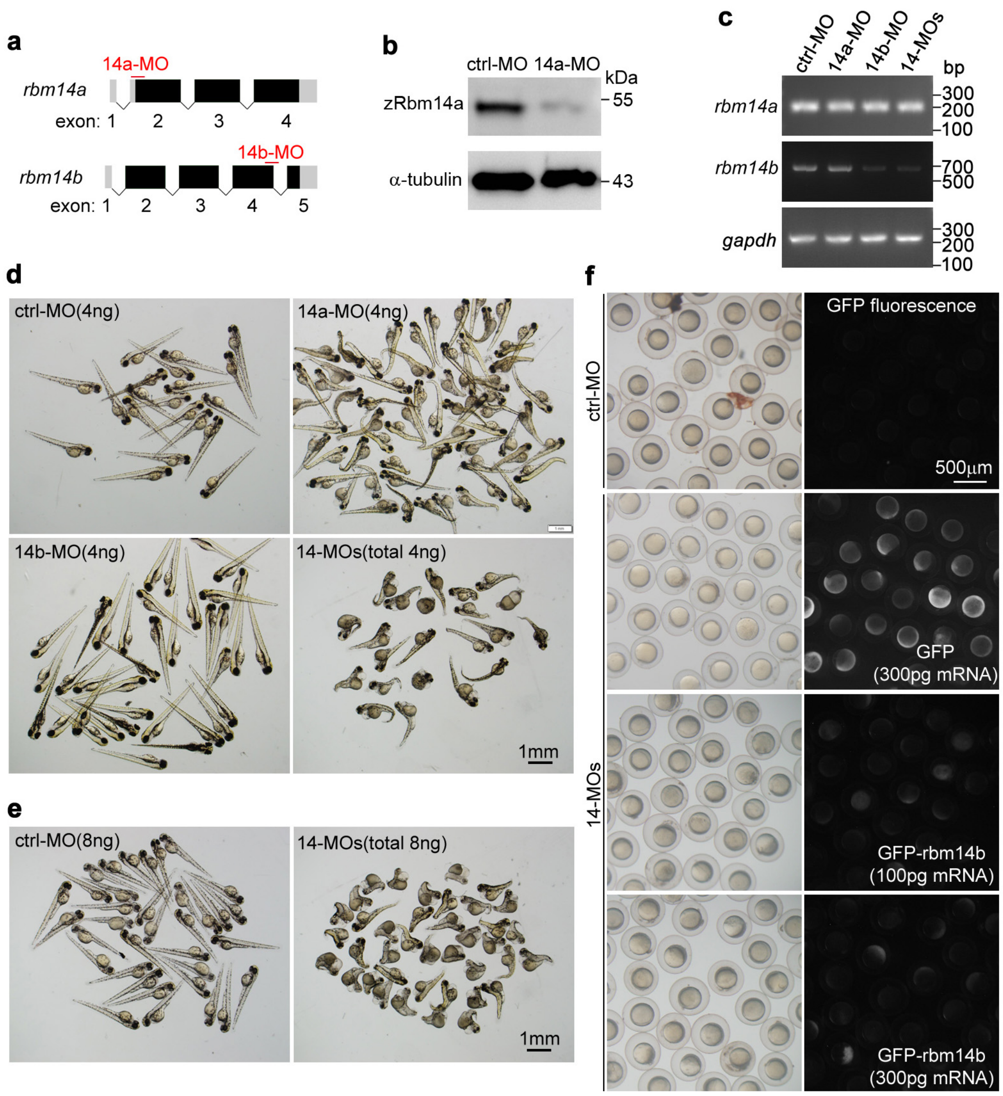

Supplementary Fig. 1 *rbm14a* and *rbm14b* are functionally redundant (related to Fig. 1). a MO targeting sites on *rbm14a* and *rbm14b*. 14a-MO was designed to block the translation initiation site of *rbm14a* mRNA. 14b-MO was designed to interrupt the *rbm14b* pre-mRNA maturation by blocking the 5' splicing site between exons 4 and 5. Coding regions are shown in black and untranslated regions (UTRs) in grey. **b** 14a-MO efficiently downregulated zRbm14a. 14a-MO or ctrl-MO was microinjected into one-cell-stage zebrafish embryo at 4 ng/embryo. Immunoblotting was performed using embryos at 24 hpf. α-tubulin served as loading control. **c** 14b-MO specifically disrupted proper splicing of *rbm14b* pre-mRNA. The indicated MOs were microinjected at 4 ng each per embryo. Total mRNAs were extracted from the embryos at 24 hpf. RT-PCRs were performed using primer pairs complementary to sequences in the exon 3 and exon 4 of *rbm14a* and the exon 4 and the 3' UTR of *rbm14b*. *gapdh* served as internal control. **d, e** 14a-MO and 14b-MO displayed a synergistic effect. Zebrafish embryos at the one-cell stage were injected with the indicated amounts of MOs and imaged at 72 hpf. **f** Expression of GFP-zRbm14b in *rbm14* morphants. The indicated amounts of mRNA were co-injected with 8 ng 14-MOs into each of the one-cell embryos. Embryos injected with ctrl-MO (8 ng per embryo) served as negative control. After the imaging at 10 hpf, GFP-positive embryos were picked out and cultured for further analysis. Note that embryo positions may drift slightly between the bright-field and fluorescent imaging.

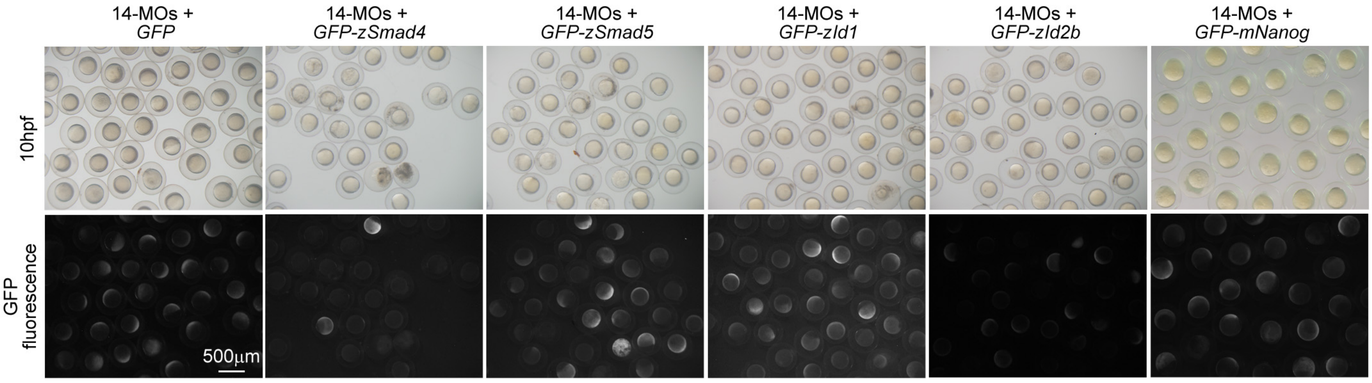

Supplementary Fig. 2 Expression of exogenous proteins in zebrafish *rbm14* morphants (related to Fig. 2). Zebrafish embryos at the one-cell stage were co-injected with 14-MOs (8 ng per embryo) and *in-vitro* transcribed mRNA (300 pg per embryo) coding for GFP or the indicated GFP fusion proteins. After the imaging at 10 hpf, GFP-positive embryos were picked out and cultured for further analysis.

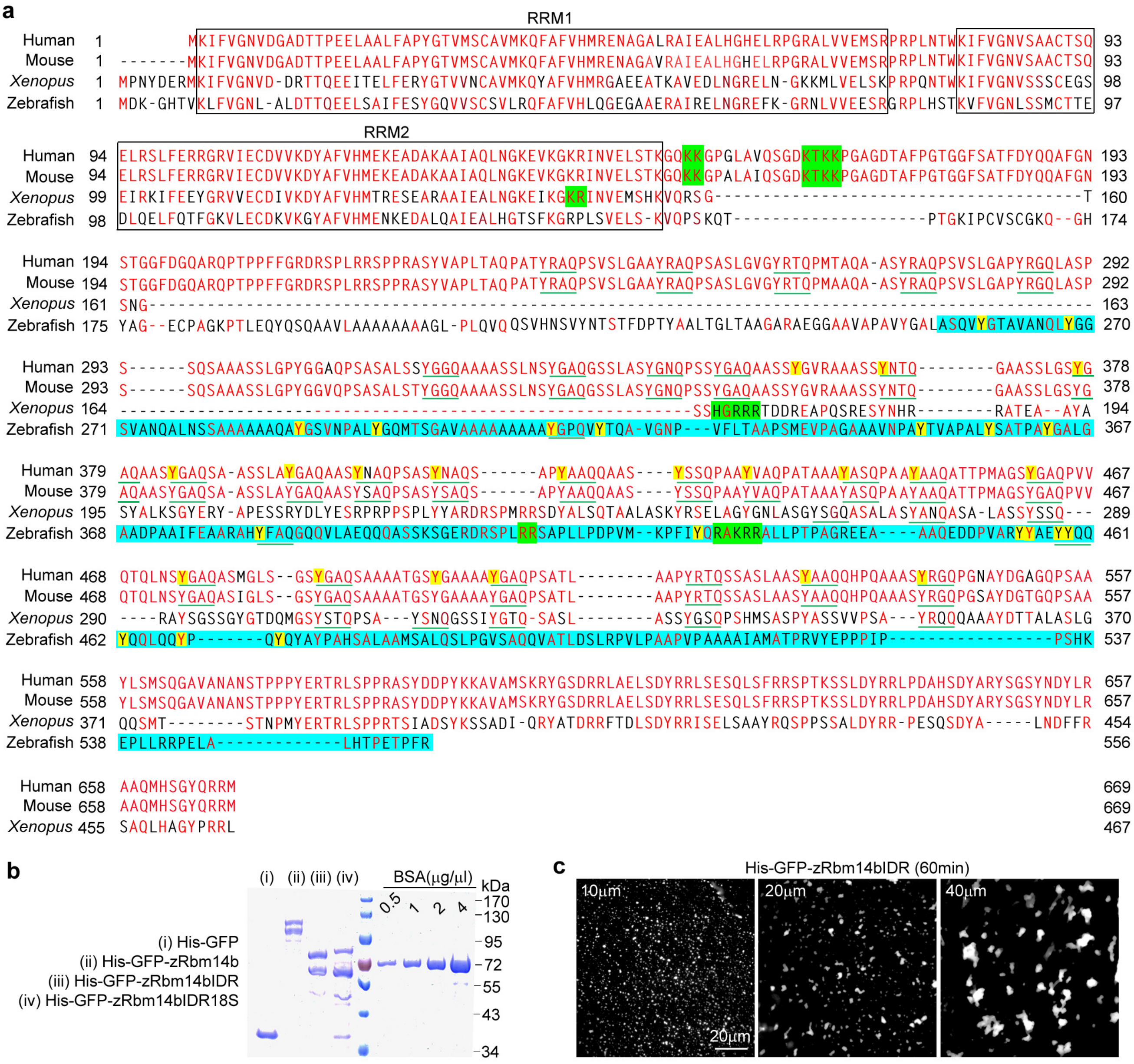

Supplementary Fig. 3 Sequence alignment of Rbm14 orthologues and protein purification for phase separation experiments (related to Fig. 3). a Sequence alignment for Rbm14 of human (GenBank accession: Q96PK6), mouse (NP_063922), *Xenopus* (NP_001079614), and zebrafish (zRbm14b; NP_997973). Amino acids conserved between any two orthologues are in red. The RRMs and putative IDR of zRbm14b are boxed and highlighted in blue, respectively. A pair of putative bipartite NLS [1] are highlighted in green for each protein. The "YXXQ" motifs are underlined. Y residues that are mutated to S to generate phase separation-defective mutants are highlighted in yellow. The documented mutation sites in human Rbm14 [2] are highlighted in yellow as a comparison. **b** Coomassie blue-staining of His-GFP-tagged zRbm14b and mutants purified from *E. coli*. The indicated amounts of bovine serum albumin (BSA) were loaded for quantitation. **c** Liquid droplet formation of zRbm14bIDR in the absence of PEG8000. His-GFP-zRbm14bIDR of the indicated concentrations was shifted from 0℃ to 25℃ for 60 min and imaged for GFP fluorescence.

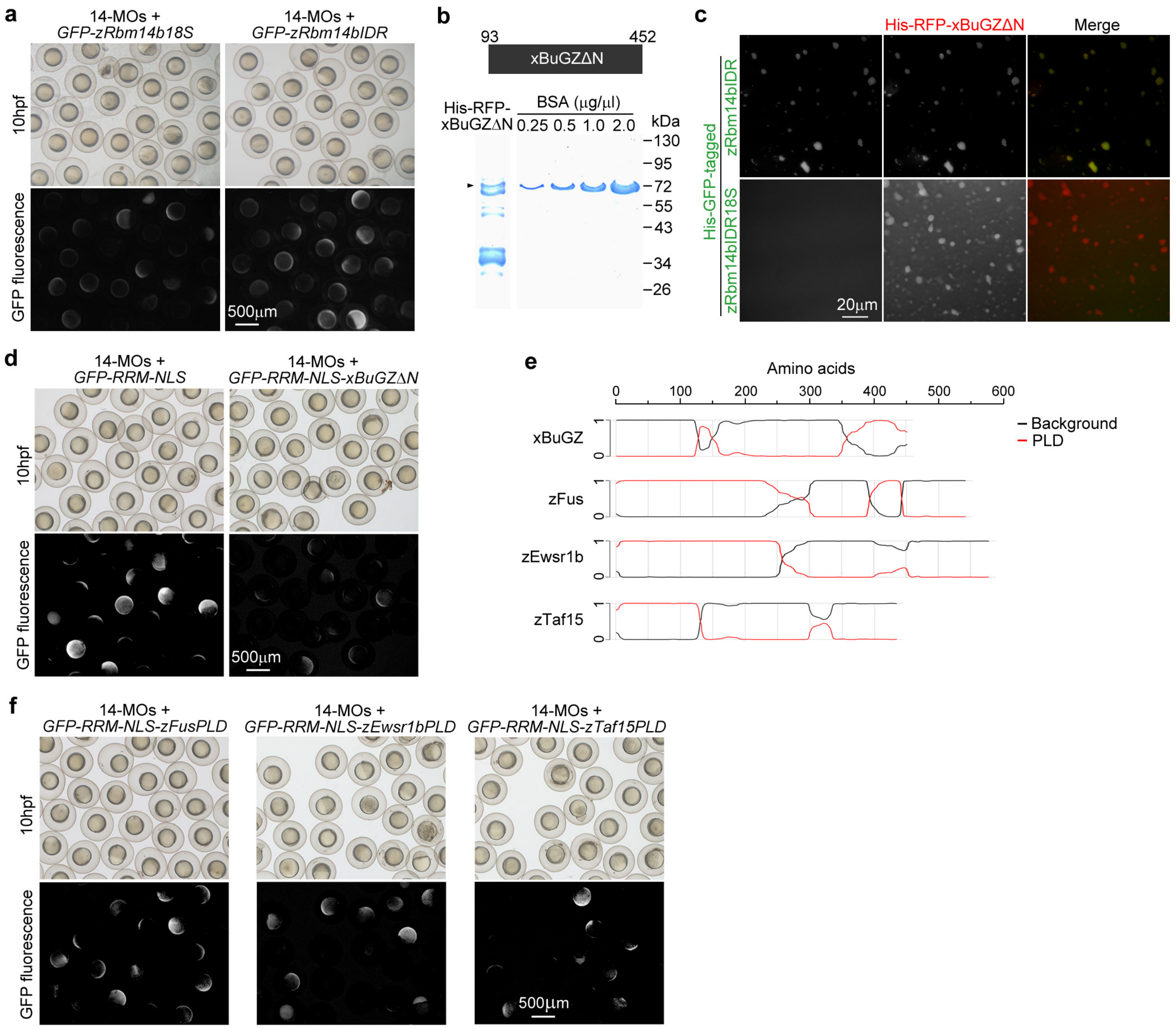

Supplementary Fig. 4 Expression of GFP-tagged zRbm14b mutants or fusion proteins in zebrafish *rbm14* morphants and co-phase separation of zRbm14bIDR with xBuGZΔN *in vitro* (related to Figs 4, 5). a, d, f Zebrafish embryos at the one-cell stage were co-injected with 14-MOs (8 ng per embryo) and the indicated i*n-vitro* transcribed mRNA (300 pg per embryo). After the imaging at 10 hpf, GFP-positive embryos were picked out and cultured for further analysis. **b** Coomassie blue-staining of His-RFP-xBuGZΔN purified from *E. coli*. The arrow indicates the position of the full-length protein. BSA was loaded for quantitation. The images were from the same gel. **c** zRbm14bIDR was able to co-phase separate with xBuGZΔN *in vitro*. Purified His-RFP-xBuGZΔN was mixed with His-GFP-zRbm14bIDR or His-GFP-zRbm14bIDR18S on ice to achieve 10-µM final concentration for each protein. The mixtures were supplemented with PEG8000 to 1% and incubated at 25°C for 5 min, followed by microscopic imaging. Note that zRbm14bIDR18S was not incorporated into the liquid droplets of xBuGZΔN. **e** PLD prediction for the indicated proteins. The diagrams were generated by using the PLAAC program (http://plaac.wi.mit.edu) [3]. Sequences with the PLD probability >0.5 (*y* axis) are considered as a PLD.

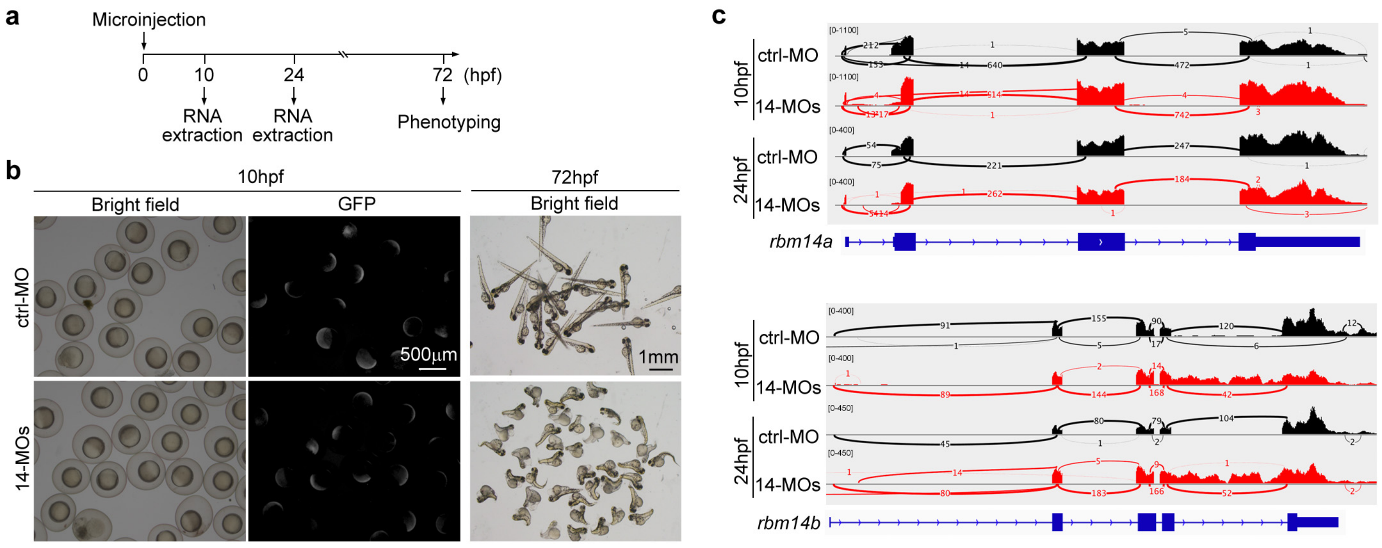

Supplementary Fig. 5 Verification of zebrafish morphants used for mRNA deep sequencing (related to Fig. 7). **a** Experimental scheme. Zebrafish embryos at the one-cell stage were injected with ctrl-MO (8 ng per embryo) or 14-MOs (total 8 ng per embryo), together with 300 pg *GFP* mRNA as injection marker. GFP-positive embryos at 10 hpf were collected for further use. 50 embryos in each group were pooled for RNA extraction. **b** Micrographs of the zebrafish embryos. **c** IGV sashimi plot of RNA-seq reads covering *rbm14a* and *rbm14b*. Please note the severe intron retention in *rbm14b* of the *rbm14* morphants.

Supplementary information accompanies the manuscript on the Cell discovery website http://www.nature.com/celldisc.

References

1 Dingwall, C. & Laskey, R. A. Nuclear targeting sequences--a consensus? *Trends Biochem Sci* **16**, 478-481 (1991).

2 Hennig, S. et al. Prion-like domains in RNA binding proteins are essential for building subnuclear paraspeckles. J *Cell Biol* **210**, 529-539 (2015).

3 Lancaster, A. K., Nutter-Upham, A., Lindquist, S. & King, O. D. PLAAC: a web and command-line application to identify proteins with prion-like amino acid composition. *Bioinformatics* **30**, 2501-2502 (2014).
